# Unraveling Quinone Degradation Enables Stabilization Using Redox Helpers in Biological and Electrochemical Systems

**DOI:** 10.1021/jacs.5c22307

**Published:** 2026-04-09

**Authors:** Shella J. Willyam, Robin A. Scullion, Sarah F. Chapman, Eleanor R. Clifford, Maxie M. Roessler, Jenny Z. Zhang

**Affiliations:** † Yusuf Hamied Department of Chemistry, 2152 University of Cambridge, Lensfield Road, Cambridge CB2 1EW, U.K.; ‡ Department of Chemistry and Centre for Pulse EPR Spectroscopy, 4615 Imperial College London, Molecular Sciences Research Hub, 82 Wood Lane, London W12 0BZ, U.K.

## Abstract

Quinones, known for their reversible redox properties, can serve as electron mediators in a wide range of contexts from electrochemical devices to biological electron transport chains. However, their practical use as redox components in aqueous environments can be significantly impaired by degradation issues. Here, we uncovered the molecular transformation mechanisms underpinning their degradation, the conditions that accelerate the degradation process, and simple strategies that can be applied to suppress their degradation. Specifically, the degradation of 2,6-dichlorobenzoquinone (DCBQ), a common electron mediator in photosynthesis and bioelectrochemistry research, was tracked under relevant operando conditions. The formation of semiquinone derivatives was identified as the key factor that drives side reactions with other molecules, including oxygen, generating deleterious radicals and decomposition pathways. These degradation pathways were accelerated under high pH conditions and in the presence of divalent cations. Guided by this mechanistic understanding, we demonstrate here that the addition of a redox helper, such as ferricyanide, establishes a redox equilibrium that effectively bypasses semiquinone buildup. This mechanism, explained by mathematical kinetics modeling, significantly prolongs the life of the quinone mediator across all tested conditions. This strategy unmasked the oxygen evolution rates of photosynthetic organisms and boosted the stability of mediated current outputs from a model living biophotoelectrochemical system, maintaining outputs at 73% higher levels over a 6 h operational period and increasing the effective half-life by 10-fold relative to control systems. These findings provide simple and effective strategies for rationally increasing the durability of quinone-based aqueous electrochemical systems, which form the essential foundation for many green energy technologies.

## Graphical abstract

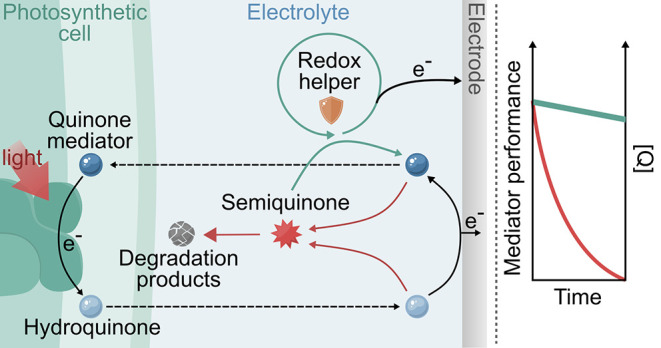

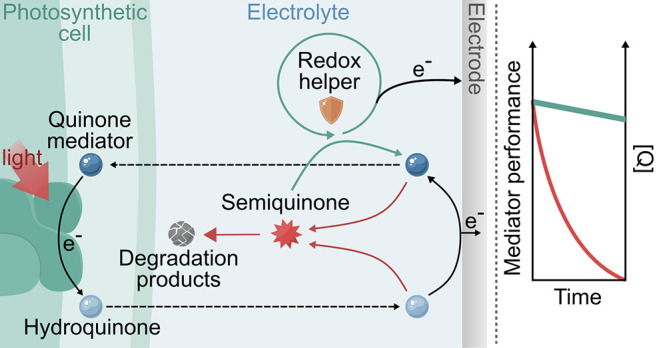

## Introduction

Quinones, characterized by the presence of two carbonyl groups attached to a conjugated six-membered ring, represent a highly versatile class of organic compounds. Their ability to undergo reversible two-electron, two-proton redox reactions coupled with their tunable redox properties, makes them excellent candidates for use in energy applications. From electrolytic water splitting to capacitive energy storage systems and redox flow batteries, quinones have demonstrated exceptional potential, scalability, and applicability in advancing sustainable energy solutions.^1–3^

Beyond energy devices, quinones play essential roles in biological processes, functioning as redox mediators within electron transport chains. For example, ubiquinone and plastoquinone pools reside within membranes, facilitating cellular respiration and photosynthesis, respectively.^4,5^ Synthetic quinones are deployed widely to substitute their biological counterparts in assays or to enhance activity. For instance, benzoquinone derivatives can be coupled with photosystem II (PSII) as powerful biomimetic electron acceptors and have enabled valuable insights into the intricate photochemical mechanisms underpinning photosynthesis.^4^

The versatility and ubiquity of quinones mean that they are well-suited for bridging biology with electrodes in the field of bioelectrochemistry. In this context, quinones have been used in biosensing and diagnostic platforms to monitor disruptions in metabolic processes with high precision.^6,7^ Furthermore, their ability to facilitate electron export from within even living cells has led to applications in bioremediation, energy generation, and biofuel synthesis.^8–11^

Among these, the use of quinones in photosynthetic biohybrid (biophotoelectrochemical) systems, particularly those utilizing photosynthetic microorganisms, has emerged as a promising technology. Unlike microbial fuel cells, which depend on organic feedstocks, photosynthetic microorganisms use sunlight as their primary energy source and water as an electron source, providing a scalable, cost-effective, and sustainable approach to energy production. Moreover, living biophotoelectrochemical systems contain self-repairing mechanisms for photosynthetic machineries, allowing them to surpass past limitations faced by isolated photosynthetic components.^12^ When coupled with synthetic quinones as redox mediators, these photosynthetic biohybrid systems can effectively channel electrons generated during photosynthesis to electrochemical circuits, achieving more than 100-fold enhancements in photocurrent outputs compared to mediator-free systems^13,14^ – pushing the performance of these biophotoelectrochemical systems closer to the predicted levels required for real-world applications.^10,15^

Despite substantial progress in understanding how the redox properties of quinones shape biological electron transfer, important gaps remain. Studies have shown that mediator’s redox potentials correlate with current generation in *Escherichia coli*, that lipophilic redox behavior can predict extracellular electron transfer in *Rhodobacter capsulatus*, and that certain quinones offer an effective compromise between algal bioelectricity and cellular toxicity.^16–18^ Even with these advances, the long-term kinetic stability of the quinones is poorly resolved. This limitation is particularly significant as synthetic quinones degrade rapidly in aqueous conditions (e.g., tetrachloro-p-benzoquinone has a half-life of slightly over 1 h at pH 7),^19^ imposing significant challenges to the scalability and long-term viability of many of these aforementioned technologies. While prior studies have shown nucleophilic attack by water as an initial degradation step, this pathway alone does not fully account for the extensive deactivation observed in quinone solutions.^20^ Uncovering the more potent degradation mechanisms is crucial for developing strategies that mitigate side reactions and ultimately enhance the long-term performance of quinone-based aqueous systems.

Herein, we unravel the transformation mechanisms governing quinone degradation in aqueous solutions and leverage these insights to develop effective stabilization approaches. Specifically, the study was conducted using DCBQ (2,6-dichlorobenzoquinone), a widely used quinone in photosynthetic and biophotoelectrochemical systems (Figure 1).^4^ Using a suite of chemical analytical techniques, we uncovered both abiotic and biotic routes of semiquinone generation from the quinone starting materials, revealing these intermediates as the key accelerant to quinone degradation. Building on this mechanistic insight, we propose intercepting these reactive intermediates as a rational stabilization principle to bypass the degradation cascade. As a proof of concept, we demonstrate that introducing ferricyanide as a water-soluble and chemically stable redox helper effectively suppresses the deleterious semiquinone intermediate, yielding a simple and versatile stabilization strategy for quinone mediators in broad contexts.

**Figure 1:**
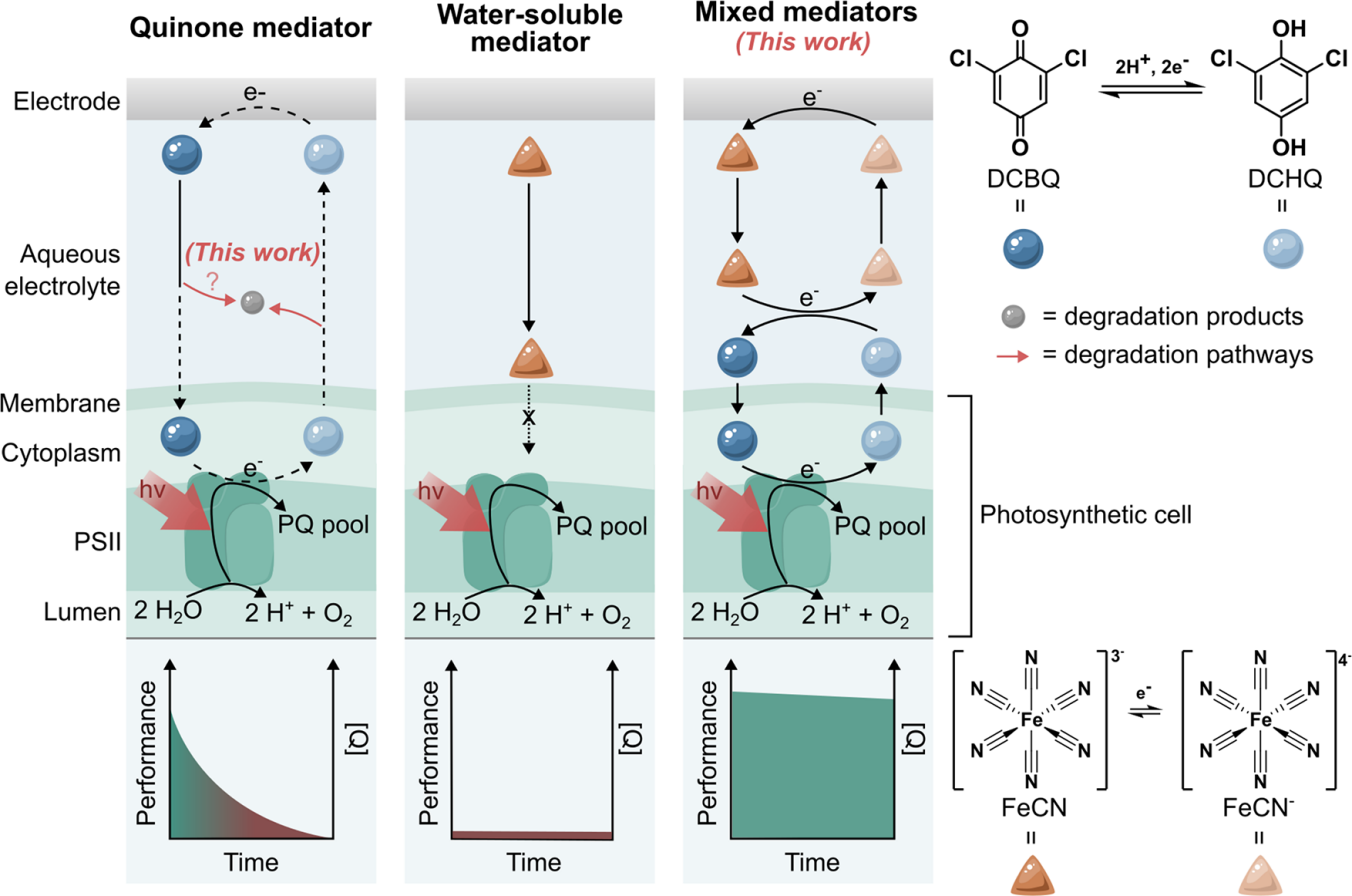
**Schematic representation of electron mediation mechanisms in a model biophotoelectrochemical system, their degradation, and an intervention strategy for benzoquinone degradation in aqueous systems.** Upon illumination, photosynthesis initiates with water oxidation at photosystem II (PSII), generating electrons that flow through the plastoquinone pool (PQ pool) and pass along the photosynthetic electron transport chain. Quinones can penetrate cell membranes and accept electrons from biocatalytic components (e.g., PSII), but they suffer from instability in water due to unclear mechanisms. The introduction of ferricyanide (FeCN) as a water-soluble redox helper that cannot enter the cells provides a straightforward strategy to enhance the stability and long-term performance of DCBQ in biotic and abiotic systems.

## Results and Discussion

### Mechanistic Study of Quinones Transformation in Aqueous Systems

To investigate the factors governing quinone degradation in water, we first examined the effect of pH. The ionic states of quinone/hydroquinone pairs and their interactions with surrounding species are strongly influenced by pH, thereby affecting their redox activity and stability. Quinones are markedly more stable under acidic conditions.^21–23^ While such acidic environments are typical for many heterotrophic bacteria, oxygenic photosynthetic organisms (e.g., cyanobacteria) and derived biophotoelectrochemical systems operate in neutral to alkaline regimes, often experiencing local alkalization during illumination due to inorganic carbon uptake. Therefore, the rest of this study focuses on this regime to assess quinone degradation under practically relevant conditions.

Quinone’s stability was evaluated by periodically recording the absorbance spectrum of DCBQ in phosphate-buffered saline (PBS), pH 5.8, 7.0, and 8.5, under dark conditions across a wavelength range of 240 to 600 nm (Figure 2b, Figure S1). At pH 5.8, DCBQ exhibited negligible degradation over the measured time frame, serving as a stable benchmark (Figure 2a). In contrast, the degradation of DCBQ was significantly faster and followed second-order kinetics with respect to DCBQ at pH 7.0 (Figure 2a), as validated by the characteristic inverse dependence of half-life on the DCBQ initial concentration (Figure S2). However, the kinetics shifted to pseudo-first-order behavior with respect to DCBQ at pH 8.5. This change indicates that pH has a crucial insight into the kinetics of the quinone degradation. At neutral pH, the concentration of the potent nucleophile OH^–^ is relatively low, which could render the initial attack on the quinone ring a significantly slow process, likely causing the kinetics to be dominated by bimolecular reactions between quinone-derived species such as the oxidation of a hydroxylated hydroquinone by another DCBQ molecule to generate semiquinones. This dependence on the concentration of two DCBQ-derived species is consistent with the observed second-order kinetics in DCBQ (Figure S2). In contrast, at pH 8.5, a significantly higher concentration of OH^–^ might make the initial nucleophilic attack more rapid. The reaction becomes pseudo-first-order because the rate is now governed by the initial nucleophilic attack of OH^–^ on DCBQ, which is first-order in DCBQ. This initial, fast step generates the intermediates that subsequently fuel the rapid semiquinone-driven degradation cascade. The absorbance of DCBQ (λ_max_ = 273 nm) declined faster at pH 8.5, with the degradation rate constant increasing approximately 5-fold compared to pH 7.0. These observations point to the involvement of hydroxide ions in the initial steps of the quinone transformation.

**Figure 2:**
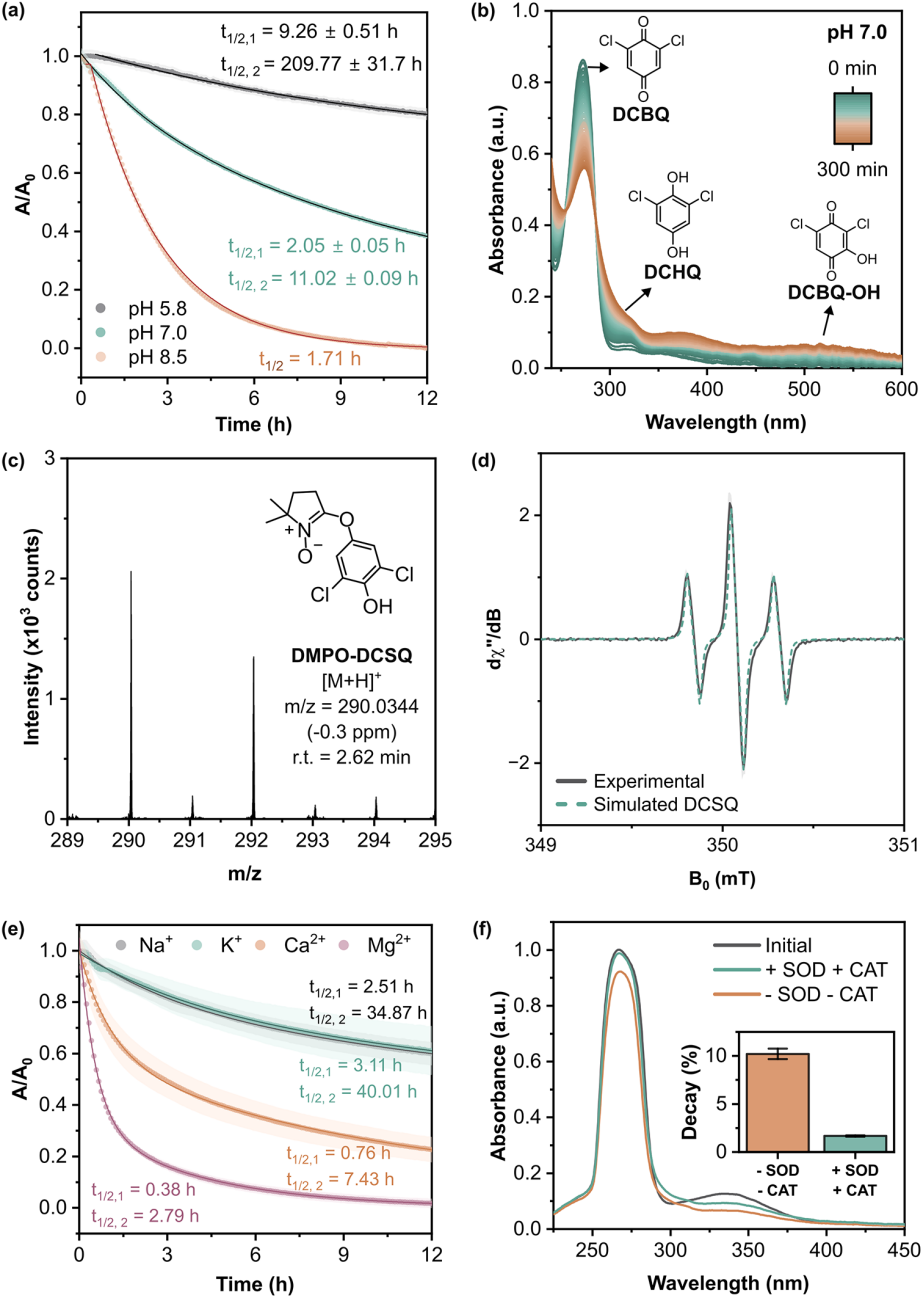
**Degradation of DCBQ in aqueous solutions. (a) Comparison of DCBQ decay kinetics under acidic (pH 5.8), neutral (pH 7.0), and basic (pH 8.5) conditions.** Half-lives (*t*_1/2_) reported are observed values for an initial concentration of 100 μM DCBQ and are dependent on the reaction order. (b) UV–vis spectra of DCBQ in PBS (pH 7.0) over 300 min. (c) ESI­(+) mass spectrum of DMPO–DCSQ adduct in DCBQ solution (solvent: HPLC-grade water). (d) EPR spectrum of DCBQ in PBS pH 7.0 at 9.8496 GHz and simulated spectrum of DCSQ (*g* = 2.0103, *A*_H1_ = 2.4 mT, *A*_H2_ = 2.4 mT). (e) Comparison of DCBQ decay kinetics in the presence of various cations with the same ionic strength. (f) UV–vis spectra of DCBQ in ethyl acetate extract after 2 h dissolution in PBS (pH 7.0). All experiments were conducted under dark conditions at room temperature. Data are reported as mean ± standard error (*n* = 3).

Simultaneously, with a decrease in DCBQ absorbance, two new peaks emerged with increasing intensity over time (Figure 2b). The peak at approximately 320 nm was attributed to 2,6-dichloro-1,4-hydroquinone (DCHQ), and a broad absorption band between 490 and 540 nm likely originates from a mixture of hydroxylated quinone derivatives, including hydroxylated DCBQ (DCBQ–OH, λ_max_ = 522 nm) and 2-chloro-6-hydroxy-1,4-hydroquinone (CHQ–OH).^24,25^ These spectral assignments were corroborated by spectroelectrochemistry and NMR and LC-MS analyses (Figures S3–S5). The formation of hydroquinones in the absence of an external donor raises the question of the source of reducing equivalents. Given the known susceptibility of quinones to photoreduction,^26^ which could theoretically be triggered by stray light or the spectrophotometer’s probe beam during prolonged assays, we performed control experiments to rule this out. White light exposure was found to have no significant effect on the reaction kinetics (Figure S6). Consequently, the reduction is attributed to a nucleophile-assisted disproportionation mechanism. One possibility is that DCBQ–OH adducts are formed via nucleophilic substitution by hydroxide ions at unoccupied carbon atoms in the quinoid ring and react with other DCBQ molecules. Another explanation is that DCHQ–OH, the product of water addition, is oxidized by other DCBQ molecules in the solution, as the electron-donating effect of the hydroxyl group lowers the redox potential. These redox processes generate hydroquinones through semiquinone radicals that could promptly react with other molecules, triggering a chain reaction that accelerates overall decomposition.

To confirm the formation of semiquinones, LC-MS analysis was conducted using DMPO (5,5-dimethyl-1-pyrroline N-oxide), which can form more stable adducts with semiquinones. Incubation of DCBQ with DMPO resulted in the detection of the DMPO–DCSQ adduct ([M + H]^+^, *m*/*z* = 290.0344, Figure 2c) and a hydroxylated-chlorosemiquinone adduct ([M + H]^+^, *m*/*z* = 272.0686, Figure S7), which is one of the intermediates of the transformation products observed (Figures 2b and S5). To definitively identify the radical intermediates, we performed electron paramagnetic resonance (EPR) spectroscopy. The spectrum obtained from the solution revealed a distinct hyperfine splitting pattern (Figure 2d) that was identified as the DCSQ radical.^27^ Control experiments confirmed that the semiquinone is generated spontaneously only upon dissolution of DCBQ in water (Figure S8). These findings provide the structural confirmation of spontaneous semiquinone formation in aqueous solutions that was previously elusive and only observed at extremely basic pH.^28^

We further assessed the contribution of semiquinones by examining the impact of cations on the DCBQ stability. The degradation rate of DCBQ increased significantly in the presence of Ca^2+^ and Mg^2+^ (Figure 2e) compared with monovalent cations (Na^+^ and K^+^) at equal ionic strength. Specifically, the half-life of DCBQ decreased by 4-fold in the presence of Ca^2+^ and 25-fold in the presence of Mg^2+^ relative to conditions with Na^+^ and K^+^. This acceleration is consistent with the known ability of divalent cations to stabilize semiquinone radical anions via complexation.^29,30^ Therefore, this stabilization shifts the equilibrium toward the formation of semiquinones, populating the reactive intermediate pool that drives an irreversible decay.

Building on these insights, we next investigated the downstream reactivity. While the reactivity of reduced quinones and semiquinones with oxygen is well-established, we identify that in quinone solutions, these reactive species are generated spontaneously through intrinsic instability rather than external reduction.^30^ Under aerobic conditions, semiquinones readily react with oxygen, forming corresponding quinones and superoxide radicals.^30^ This interaction is evidenced by the significantly higher semiquinone concentration observed under anaerobic conditions compared to that under aerobic conditions (Figure S9). This process further accelerates quinone decomposition and generates reactive oxygen species (ROS). This conclusion is supported by the detection of DMPO–OH radical adduct in aerobic DCBQ + DMPO solutions (Figure S10),^27,31,32^ which is consistent with the trapping of ROS such as superoxide or singlet oxygen that can yield DMPO–OH as a stable product in the presence of DMSO.^33,34^ No distinctive EPR signal for the DMPO-semiquinone adducts was observed, indicating that any product of the direct reaction between DMPO and semiquinones does not produce paramagnetic species. This suggests that these adducts are oxidized by other quinone molecules to form EPR-silent species detected using LC-MS (Figure 2c, Figure S7).

While anaerobic isolation would preclude this pathway, this approach fails to address the operational reality of many bioelectrochemical systems, most notably in systems involving oxygenic photosynthesis. Therefore, we evaluate the contribution of oxygen by comparing DCBQ stability in the absence and presence of ROS scavengers catalase (CAT) and superoxide dismutase (SOD). Our results show a marked reduction in DCBQ degradation of 78% over 2 h in the presence of these scavengers (Figure 2f). SOD catalyzes the dismutation of superoxide formed by semiquinones and oxygen into hydrogen peroxide, which is subsequently decomposed into water and oxygen by CAT. Therefore, the addition of SOD and CAT shifts the reaction equilibrium toward quinone formation and mitigates the degradation process. This finding confirms the pivotal role of semiquinones and the ROS formed in the degradation pathway.

Synthesizing our findings with existing literature, we propose the primary pathways of quinone transformation in aqueous solutions in Scheme 1. Semiquinones are produced either through the partial oxidation of hydroxylated hydroquinones or via reactions between quinones and unstable OH-quinone adducts.^20,28^ These semiquinones subsequently react with other species in the solution, including oxygen, leading to further degradation. This mechanistic insight opens the possibility of preserving quinone stability in aqueous solutions by strategically intervening at specific stages in the degradation pathway, thereby mitigating the adverse effects of semiquinones and ROS.

**Scheme 1:**
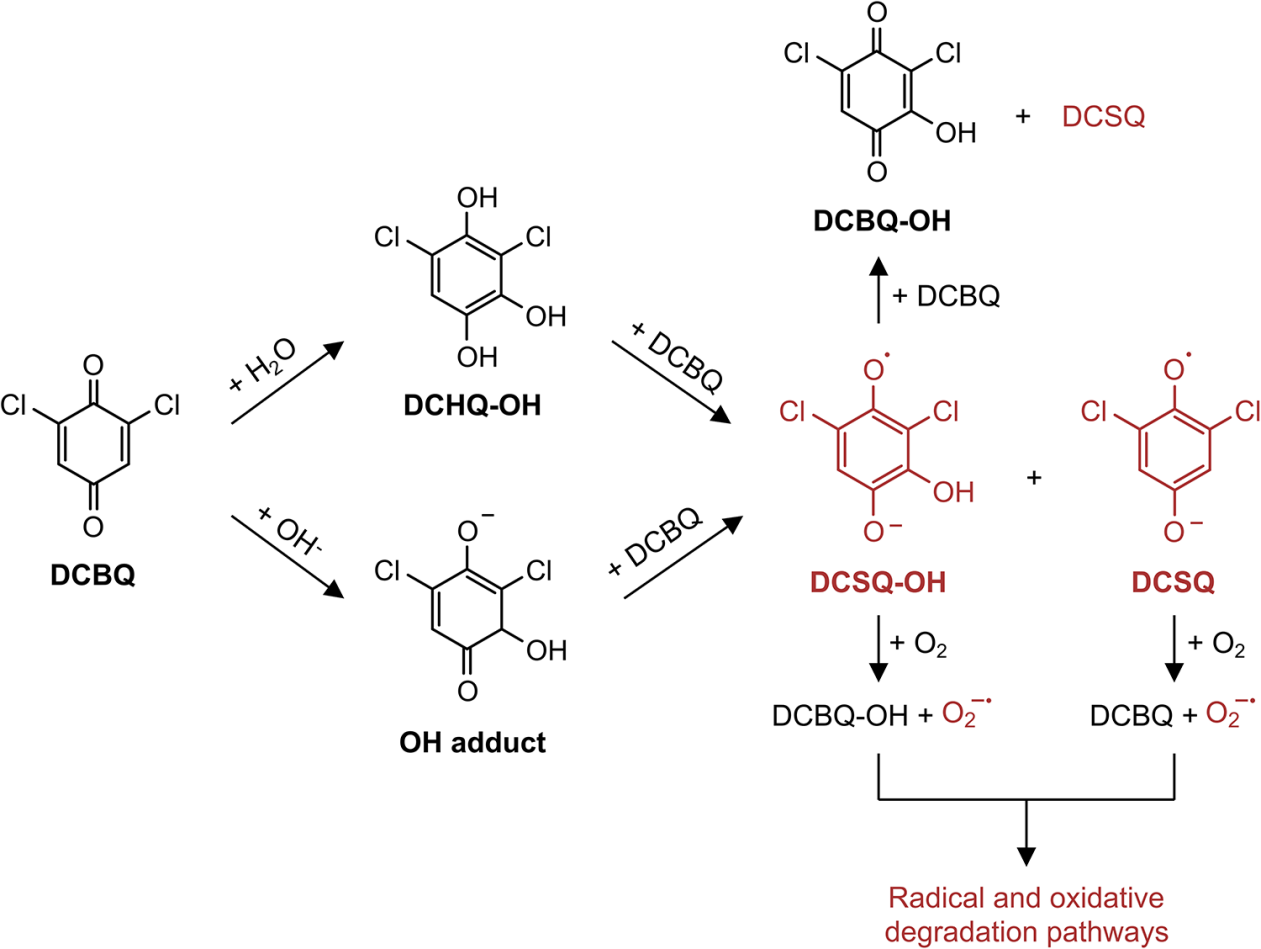
**Proposed Pathways for DCBQ Transformation and Degradation in Aqueous Solutions**. Quinones undergo nucleophilic attack and interact with other quinone molecules, leading to the formation of unstable semiquinones; these semiquinones readily undergo side reactions and degradation, highlighting their pivotal role in the overall transformation process.

### Enhanced Quinone Stability in the Presence of a Redox Helper

Based on the mechanistic insights gained, a strategy was explored to enhance quinone stability by adding a redox helper to bypass harmful intermediates. Thermodynamically, this strategy relies on the helper acting as an electron sink; thus, it must possess a reduction potential sufficiently positive relative to that of the semiquinone couple to drive spontaneous oxidation. Ferricyanide (*E*_m_ = 0.42 V vs SHE) was chosen as a candidate that meets this requirement. It is a well-characterized redox molecule capable of oxidizing DCBQ (*E*_m_ = 0.32 V vs SHE) and has been used in combination with DCBQ in many studies to enhance the performance of PSII.^35,36^ However, the specific mechanism underlying this enhancement has been attributed to the prevention of electron recombination, which enables maximum PSII activity.^37,38^ Based on our new understanding of the degradation pathways, we instead hypothesize that ferricyanide can efficiently oxidize any semiquinones present in the solution to enhance the stability and performance of the DCBQ.

To test this hypothesis, the degradation of DCBQ must be monitored in real time in the presence of ferricyanide. This was a significant challenge using common techniques, including UV–vis spectroscopy and cyclic voltammetry, due to the overlapping signals of the quinone and ferricyanide species, along with their reduced species. Following considerable optimization efforts (see Supplementary Notes for optimization details), this was achieved using square wave voltammetry (SWV) performed at 5 min intervals. SWV was ultimately selected for its ability to resolve the distinct reduction peaks of DCBQ (−0.1 vs −0.2 V vs SHE) and ferricyanide (0.2–0.4 V vs SHE), allowing their peak currents to serve as proxies for concentration. PBS was replaced with carbonate-buffered saline to minimize the electrode passivation and ensure the signal stability. Consequently, the pH was increased to 9.0 to match the effective buffering range of the carbonate system. Representative SWV traces collected at pH 9 in the absence and presence of ferricyanide are shown in Figure 3a,b (see SWV traces at pH 7 in Figure S11), while average peak currents of DCBQ at different time points are presented in Figure 3c for pH 9 and Figure SN2 for pH 7.

**Figure 3:**
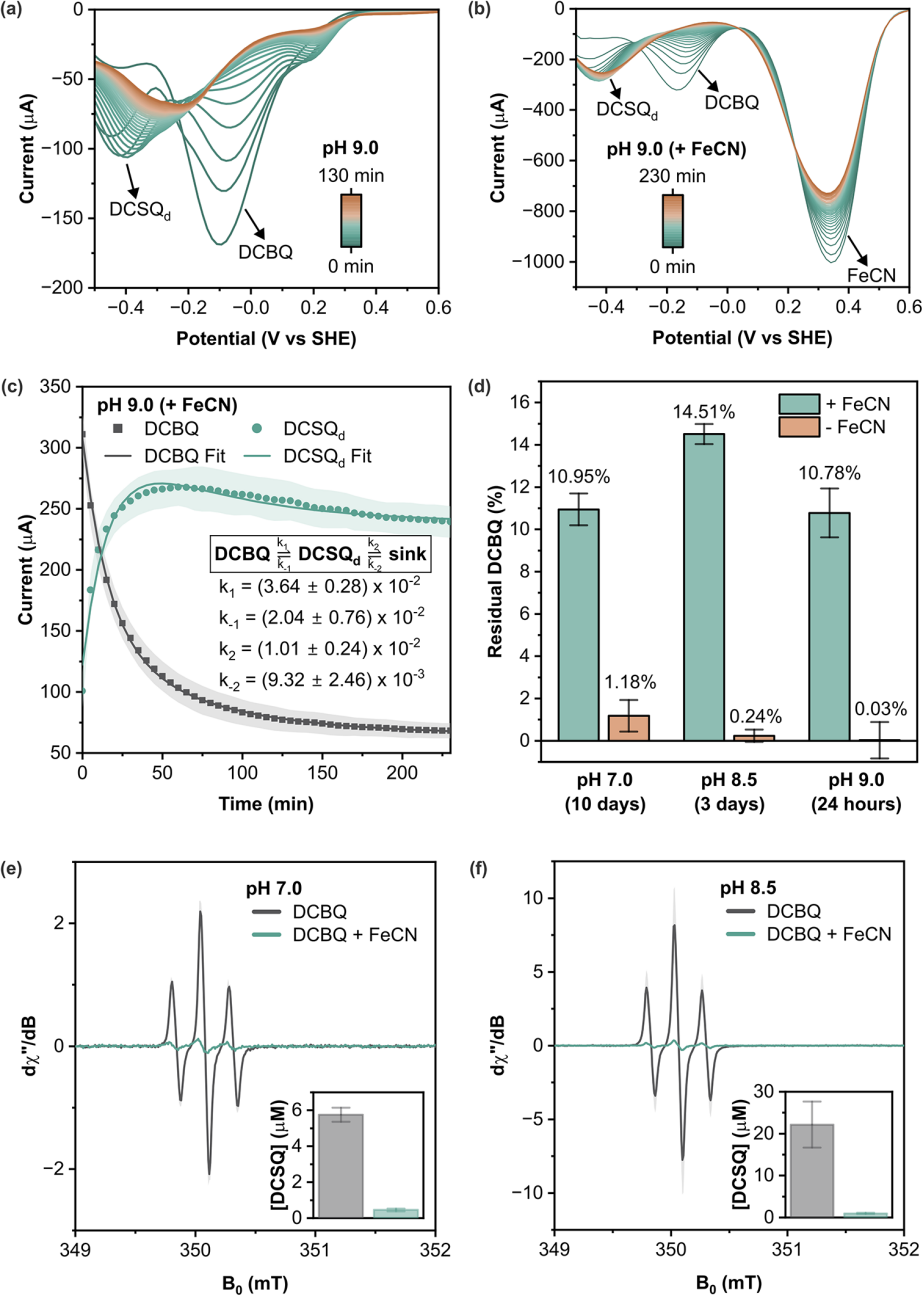
**Analysis of DCBQ degradation kinetics using square wave voltammetry (SWV).** Representative SWV curve of DCBQ recorded in carbonate-buffered saline pH 9.0 over time (a) without and (b) with ferricyanide (FeCN) ([DCBQ] = 500 μM, [FeCN] = 2.5 mM). (c) SWV peak currents as a proxy of DCBQ concentration over time in carbonate-buffered saline pH 9.0 in the presence of ferricyanide, fitted to curves modeled by ordinary differential equations (see Supplementary Notes for details). (d) Comparison of residual DCBQ concentrations with and without FeCN at the final time points for each buffer condition (PBS pH 7.0 and 8.5; carbonate-buffered saline pH 9.0). The complete degradation profiles over time are shown in Figure S16. Data are reported as mean ± standard deviation (*n* = 3). EPR spectra of DCBQ in PBS pH (e) 7.0 and (f) 8.5 in the absence and presence of FeCN at 9.849 GHz, along with the calculated DCSQ concentration.

At pH 7, the degradation of DCBQ in carbonate buffer could be fit to either first-order or second-order kinetics with respect to DCBQ (Figure SN2c; see Table SN1 for all fitting parameters). This intermediate reaction order contrasts with the second-order kinetics observed in PBS at the same pH (Figure 2a). A possible explanation is that in a carbonate buffer, interactions between semiquinones and CO_2_ shift the reaction toward the formation of semiquinones.^39^ This is supported by a substantially faster degradation rate compared with PBS (Figure 2a and Figure SN2c). The half-life of DCBQ in carbonate buffer was calculated to be 57.7 ± 8.7 min, whereas when ferricyanide was present, the half-life significantly increased to 159.7 ± 50.4 min, showing a 2.8-fold enhancement in stability (Figure SN2c; see Table SN1 for fitting parameters). These results imply that ferricyanide bypasses the initial process by rapidly reoxidizing the semiquinone, thereby establishing a dynamic redox equilibrium that slows overall degradation.

At pH 9, the degradation kinetics of DCBQ become considerably more complex due to the favored formation of OH adducts over DCSQ–OH (Scheme 1) and because the reaction with OH^–^ is faster than that with water. A simple exponential decay model proved to be insufficient to capture the observed dynamics, especially in the presence of ferricyanide (see Supplementary Notes for details). Therefore, the degradation was interpreted using a simplified model (Figure 3c, Figure SN5c) based on Scheme 1. In this model, it is hypothesized that the subsidiary peak observed at around −0.4 V (Figure 3a,b) with and without ferricyanide reflects the concentration of redox-active intermediates, likely DCSQ derivatives (hereafter DCSQ_d_). DCBQ is initially converted into DCSQ_d_, which subsequently transforms into a pool of degradation products termed the “sink” (Figure 3c and Figure SN5c, see Supplementary Notes for the model equations). It is assumed that the reaction rates are such that DCBQ remains in excess relative to either DCHQ–OH or the OH-adduct formed in the initial step, allowing the first two reaction steps to be effectively treated as a single kinetic process.

In the absence of ferricyanide, the reverse rate constants for both the formation of DCSQ_d_ and its conversion to sink are negligible, resulting in a predominantly unidirectional degradation pathway (Figure SN5c; see Table SN2 for fitting parameters). However, when ferricyanide is present, dynamic equilibria are established in both steps; the reverse rate constants become comparable in magnitude to the forward rate constants (Figure 3c; see Table SN2 for fitting parameters). The stabilization effect was also observed in neutral PBS and BG11 media, common media rich in cations and other nutrients used in biophotoelectrochemistry (Figure S12). This indicates that ferricyanide actively reoxidizes the semiquinone intermediates and their subsequent derivatives back to DCBQ, thereby shifting the equilibrium toward quinone retention. The temporal change of DCSQ radical concentration measured using EPR (Figure S13) shows excellent agreement with the trends predicted by the kinetic model and the SWV data (Figure 3c and Figure SN5). Furthermore, this mechanism was directly validated by quantifying the steady-state concentration of semiquinone radicals using EPR spectroscopy (Figure 3e–f). In the absence of ferricyanide, a substantial accumulation of DCSQ radicals was observed at both pH 7.0 and 8.5. However, upon the addition of ferricyanide, the semiquinone signal was drastically suppressed, confirming that the redox helper effectively intercepts these reactive intermediates before they can drive the degradation cascade. This redox mechanism is also reflected in the change of ferricyanide absorbance (Figure S14) and is supported by the higher abundance of the DMPO–DCSQ adduct (i.e., DCSQ_d_) observed in the presence of ferricyanide, alongside the reduced formation of DMPO–CSQ, one of the degradation products (i.e., sink) (Figure S15). Although the fitted curves for DCSQ_d_ vary slightly from the experimental data (Figure 3c, Figure SN5c), this discrepancy may be attributed to experimental error and the assumption that the concentration of the sink remains constant, which is likely unrealistic. Under these conditions, the model predicts that the steady-state concentration of DCBQ stabilizes at approximately 20% of its initial value.

In support of this prediction, a longer-term quantification (Figure 3d) demonstrates that DCBQ is markedly stabilized by ferricyanide under both neutral and basic conditions. The long-term degradation profiles (Figure S16) show the decay rate slowing significantly over time, with the residual DCBQ concentration remaining relatively stable, consistent with the kinetic model’s prediction of an approach to a steady state. The final residual DCBQ concentrations (summarized in Figure 3d) highlight the magnitude of the protective effect of the redox partner. After 10 days at pH 7.0, the residual DCBQ concentration was 10.95% with ferricyanide compared to just 1.18% in its absence, showing a 9.3-fold improvement in stability. The effect was even more pronounced under alkaline conditions. After 3 days at pH 8.5, the stabilized sample retained 14.51% of DCBQ, whereas the control was nearly completely degraded (0.24%). Similarly, after 24 h in carbonate buffer pH 9.0, 10.78% of DCBQ remained with ferricyanide compared to complete degradation in the control. Nevertheless, slow, gradual decay persisted, especially under higher pH; for instance, DCBQ concentration in carbonate buffer pH 9.0 still declined from 10.78% after 24 h to 4.75% over 9 days despite the use of ferricyanide. This may be attributed to three converging factors: (1) kinetic competition, where a finite fraction of semiquinones inevitably escapes interception by ferricyanide; (2) stoichiometric depletion, where the gradual consumption of the oxidant (ferricyanide) reduces the thermodynamic driving force; and (3) downstream reactions of the sink, which are not accounted for in the current kinetic model. Therefore, while the redox helper strategy may not entirely prevent degradation, it shifts the bottleneck to these slower secondary pathways, providing a significant stability enhancement that can be further improved for more robust systems. This establishes continuous electrochemical regeneration of the redox partner as a critical design requirement to counteract depletion over time while underscoring the value of structural tuning to further minimize semiquinone formation.

### Preserving Quinone Redox Activity in Biological and Bioelectrochemical Systems

Having demonstrated that a redox helper effectively enhances quinone stability in abiotic systems, we sought to translate this strategy to more complex biologically relevant conditions. Successful implementation in biotic systems requires adherence to two additional design principles. Structurally, the redox helper should have relevant spatial compartmentalization; for example, in this case, it should remain membrane-impermeable to preclude interference with intracellular electron transport chains or additional toxicity. Chemically, its reduction potential must be bounded to prevent nonspecific oxidative damage to cellular components. With these criteria, we evaluated the strategy under biological systems, where the presence of oxygen and the dynamic biological processes present further challenges to the quinone mediator stability.

Oxygen, present in both the environment and biological systems, can act as an electron acceptor and accelerate quinone degradation, as previously discussed (Scheme 1). We further tested the effectiveness of using a redox helper in oxygenated environments by employing *Synechocystis* sp. PCC6803 (hereafter referred to as *Synechocystis*) as our model photosynthetic microorganism. Photosystem II (PSII) is a membrane-bound protein complex that harnesses light energy to oxidize water molecules, liberating electrons and oxygen as byproducts. DCBQ has been shown to permeate into *Synechocystis* cells and accept electrons from PSII, thereby enhancing PSII turnover and boosting oxygen production during photosynthesis (Figure 4a).^12,40^ By observing the changes in oxygen levels within the cell suspension under alternating light and dark cycles, we assessed the effectiveness of this stabilization strategy under conditions that closely mimic those encountered during photosynthesis.

**Figure 4:**
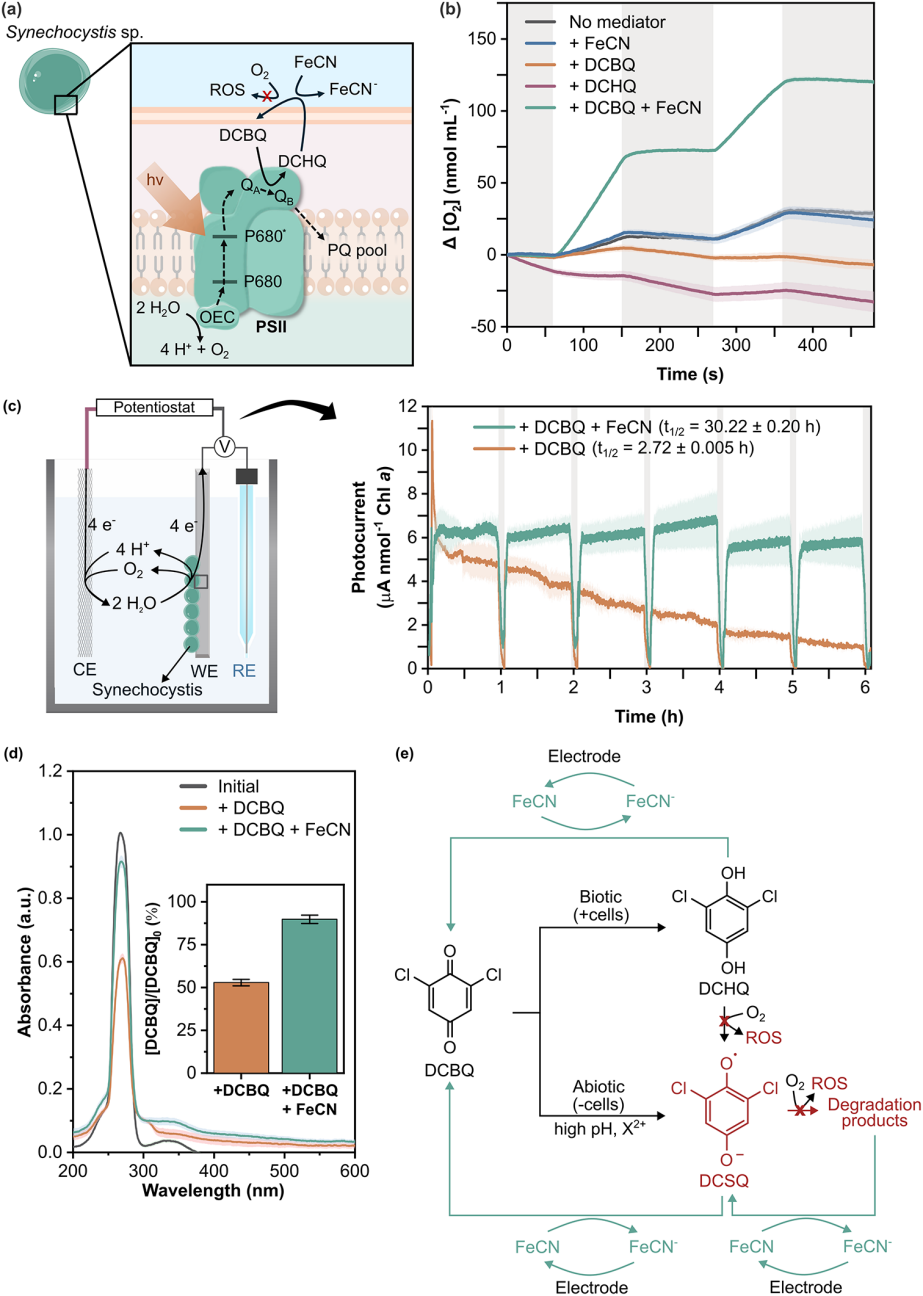
**Impact of ferricyanide on the stability of quinone-mediated biological and bioelectrochemical systems.** (a) Illustration depicting the electron transfer process from PSII to DCBQ in *Synechocystis* and the role of ferricyanide (FeCN) in mitigating oxygen consumption caused by DCHQ. FeCN can be reversibly reduced into ferrocyanide (FeCN^–^). OEC: oxygen-evolving complex, PQ: plastoquinone, PSII: photosystem II, Q_A_/Q_B_: PSII terminal electron acceptors, and ROS: reactive oxygen species. (b) Temporal changes in oxygen concentration in *Synechocystis* suspensions with various electron acceptors at 25 °C. Gray and white shades indicate dark and light periods, respectively. (c) Schematic illustration of the biophotoelectrochemical setup and the resulting DCBQ-mediated photocurrents stemming from the photosynthetic machineries in the absence and presence of FeCN. White and gray shades indicate light and dark conditions, respectively. Light conditions: 680 nm, 1 mW cm^–2^, cycle of 55 min on and 5 min off. Applied potential: 0.4 V vs Ag/AgCl (sat. KCl). Temperature: 25 °C, electrolyte: PBS pH 7.0. CE: counter electrode, RE: reference electrode, and WE: working electrode. Half-lives (*t*_1/2_) were obtained by fitting the photocharge at different time points to a first-order exponential equation. (d) Quantification of the remaining DCBQ amount after 2 h of biophotoelectrochemical experiment. (e) Role of ferricyanide in suppressing DCBQ degradation caused by biotically (i.e., by accepting electrons from photosynthetic electron transport chain) and abiotically formed semiquinones in bioelectrochemical systems based on the mechanistic study. Data are reported as mean ± standard error (*n* = 3 biological replicates). [DCBQ] = 200 μM, [FeCN] = 1 mM for all experiments.

Contrary to expectations, Figure 4b shows that photosynthesis in the presence of DCBQ did not lead to an increase in oxygen production. The measured oxygen levels represent a net change resulting from the dynamic balance between gross production from PSII and various consumption pathways. A more rapid oxygen depletion in the dark was followed by a slower rise in the subsequent light phase, suggesting that the overall consumption intensified over the course of the experiment. Exogenous mediators are known to alter biological oxygen fluxes; for example, ferricyanide decreases gross oxygen evolution via the State 2 transition and reduces biological oxygen uptake by outcompeting Mehler-like reactions.^41^ Because DCBQ accepts electrons mostly from PSII,^12,40^ it likely maintains a more oxidized plastoquinone pool and promotes a State 1 transition. This biological shift, combined with the suppression of endogenous oxygen-consuming sinks, theoretically increases the net oxygen levels during light periods significantly. Therefore, the observed severe oxygen depletion in cells supplemented with DCBQ suggests that abiotic chemical consumption completely overshadows these physiological dynamics. This abiotic sink arises directly from the conversion of DCBQ to hydroquinones and semiquinones in the suspensions. To further investigate this phenomenon, analogous measurements were conducted using cell suspensions supplemented with chemically synthesized DCHQ (Figure 4b). The results revealed a significantly faster decrease in oxygen concentration compared with other conditions. This trend was also evident in abiotic DCBQ and DCHQ solutions (Figure S17), confirming that semiquinones and hydroquinones react directly with oxygen.

The introduction of ferricyanide into the suspensions effectively mitigated these side reactions, thereby unveiling the concealed photosynthetic oxygen evolution (Figure 4b). By effectively shunting deleterious side pathways, this practice enhances the accuracy and reliability of the experimental results of photosynthetic oxygen measurements.

Empirically, the combination of quinone and ferricyanide has been observed to give rise to maximum PSII turnover. Subsequently, they are sometimes delivered together in oxygen evolution assays,^5,42^ but this is not standard practice.^43–45^ This study provides a mechanistic explanation for this observation, enabling the rational design of the best practice. Beyond aiding in the regeneration of quinones, the redox helper serves a critical protective role by reducing the side reactions with oxygen and mitigating oxidative damage caused by semiquinones and ROS. In the absence of electrochemical regeneration, an excess of the helper is needed, as it functions as a stoichiometric oxidant. Our kinetic calculations confirm that the thermodynamic driving force is maintained without limiting depletion over the course of experiments. By effectively suppressing deleterious side pathways, this practice enhances the accuracy and reliability of the experimental results of photosynthetic oxygen measurements.

The strategy was further extended to a biophotoelectrochemical system. In a model system where cyanobacteria *Synechocystis* serve as the source of photosynthetic electrons, DCBQ acts as an electron shuttle connecting the photosynthetic machineries performing water oxidation inside the cells to the electrode – with the output being enhanced anodic photocurrents. The *Synechocystis* cells were immobilized on inverse-opal indium tin oxide electrodes, and the photocurrent outputs were monitored by using chronoamperometry (Figure 4c). Experiments were conducted in PBS electrolyte (pH 7) at an applied potential of +0.4 V vs Ag/AgCl (sat. KCl) and under 680 nm illumination (1 mW cm^–2^), a standard light condition for these systems.^13,40^ Stirring was applied to minimize diffusional limitations.

Figure 4c shows that the photocurrent outputs from DCBQ-mediated systems decreased exponentially with a half-life of 2 h. Previous studies have linked similar photocurrent decay to the cytotoxicity of mediators and photoinactivation.^11,40,46^ These factors are unlikely in this experiment; *Synechocystis* are known to remain highly active in such systems for over 5 days,^12^ and the concentration of DCBQ used was observed to be nontoxic to *Synechocystis*.^40^ It is most probable that the degradation of DCBQ is the key accelerant to the observed decrease in photocurrent outputs. Although the major transformation products, hydroxylated quinones/hydroquinones, are expected to remain redox-active, they exhibit significantly lower lipophilicity at neutral and intracellular pH levels, reducing their ability to travel across membranes and perform as diffusible mediators (Figure S18).^47^ To verify that the decreased photocurrent was mainly due to DCBQ degradation, the residual DCBQ concentration in the electrolyte was measured after 2 h of experimentation (Figure 4d). The amount of DCBQ in the bioelectrochemical cell dropped by 48% over this period, closely mirroring the reduction observed in photocurrent outputs. In abiotic solutions, the decrease was only 37% over the same period (Figure S19). This additional loss in the presence of cells suggests that cell-associated processes, such as intracellular sequestration or metabolic degradation, likely contribute to DCBQ loss in the bulk electrolyte,^46^ although the precise biotic mechanisms remain to be elucidated. These results confirm that the chemical instability of DCBQ is the principal factor limiting the operational lifetime of the system.

Upon adding ferricyanide into the system, the photocurrent outputs remained relatively high and stable over 6 h (Figure 4c), with an extrapolated half-life of 30.22 h. While ferricyanide cannot pass through the plasma membrane of cyanobacteria (Figure 1), it can cross the outer membrane and accept electrons from the plasma membrane respiratory chain.^48,49^ However, control experiments with ferricyanide alone confirmed that its direct contribution to the observed signals is minimal. In oxygen evolution measurements, the net oxygen evolution rates in the presence of ferricyanide remained comparable to those in the control (Figure 4b). Similarly, the increase in photocurrents generated by ferricyanide alone (Figure S20) was negligible compared to the microampere currents observed with DCBQ (Figure 4c). Thus, although a minor fraction of electrons may be accepted directly from the plasma membrane, the predominant photocurrent observed in dual-mediator systems stemmed from electrons relayed by the photosynthetic reaction centers to DCBQ. The electrons are then either transferred to ferricyanide and subsequently to the electrode, or directly from DCHQ to the electrode. Despite continuous stirring, the heterogeneous two-step electron transfer kinetics between DCHQ and the electrode is slower than homogeneous kinetics between DCHQ and ferricyanide. Therefore, ferricyanide helps in mitigating the formation of semiquinones both in solution and during the oxidation of hydroquinones on the electrode surface (Figure 4e). This is supported by the substantially smaller decrease (10%) in DCBQ concentration observed after 2 h of experiment when ferricyanide was present (Figure 4d). In contrast to the isolated biological assay, the working electrode continuously regenerates ferricyanide, enabling the helper to function catalytically.

Ideally, the redox helper should also be robust. In the studies here, ferricyanide remains stable over experimental time scales since the use of 680 nm irradiation avoids spectral overlap with the UV-blue absorption bands of ferricyanide and results in no significant photodegradation compared to dark controls (Figure S21). However, ferricyanide is used as a demonstration model redox helper for our biological and electrochemical model systems; each application must select redox helpers that are robust in their unique contexts.

## Conclusion

In summary, this study uncovered the transformation mechanisms governing quinone degradation in aqueous systems, identifying semiquinone radicals as the critical species that rapidly engage in a cascade of autocatalytic reactions, contributing significantly to the irreversible decay of quinones. These radicals can be generated not only spontaneously in water but also through the acceptance of electrons from the biological electron transport chains. This mechanistic understanding suggests that quinone stability is highly sensitive to environmental conditions and can be enhanced by controlling the conditions. For instance, degradation is approximately 5-fold slower at neutral pH compared to alkaline conditions. Stability is also dramatically improved by avoiding divalent cations – the presence of Mg^2+^ accelerated degradation 25-fold compared to monovalent cations. Furthermore, the addition of ROS scavengers reduced degradation by 78%, confirming that managing oxygen and its radical byproducts is a viable stabilization pathway.

While these environmental controls are effective, they are often incompatible with the operational constraints of many practical systems. To overcome these limitations, we introduce a redox mediator helper, specifically ferricyanide, to establish a redox equilibrium that suppresses the occurrence of semiquinone. This strategy preserves quinone redox functionality, particularly in biological systems, which must operate within neutral/alkaline pH ranges or media rich in divalent cations and oxygen. This protective effect was consistently observed under a variety of conditions, ranging from neutral to alkaline pH environments as well as in complex, cation-rich media, achieving up to a 9.3-fold improvement in long-term stability. Moreover, when DCBQ was combined with ferricyanide as the model mediator helper in cyanobacterial and biohybrid systems, the strategy revealed the true oxygen evolution output, boosting the measured rate by nearly 6-fold compared to DCBQ alone. This significant discrepancy reveals a systematic oversight in many oxygen evolution protocols, implying that photosynthetic rates reported in studies without the use of a redox helper may be systematically underestimated due to parasitic oxygen consumption. Similarly, this may confound other oxygen-sensitive quinone-mediated assays such as mitochondrial respiration and enzymatic kinetics studies. In addition, photocurrent outputs were 73% higher after 6 h, and the half-life was extended 10-fold compared to those with just DCBQ, underscoring the viability of this strategy for both biological and electrochemical applications. While ferricyanide serves here as an effective model to demonstrate this mechanistically guided stabilization strategy, future applications may benefit from the design of more robust partners to ensure long-term stability.

In a broader context, the new understanding of quinone degradation and stabilization pathways gained from this study establishes a fundamental framework for rational mediator design. While we demonstrated the efficacy of the redox helper strategy here, the identification of the semiquinone bottleneck opens avenues for diverse engineering solutions, such as molecular modification or alternative scavenger systems. Consequently, these findings provide a versatile toolkit for advancing a wide variety of sustainable technologies that require robust aqueous redox chemistry. By resolving fundamental instability issues, this work paves the way for more efficient redox-mediated systems, ranging from batteries and homogeneous catalysts to electrochemical carbon capture and semiartificial photosynthesis.

## Methods

All materials and chemicals used in this study were purchased from the indicated commercial suppliers and used as received, unless specified otherwise. Water was filtered using a Millipore Milli-Q water purifying system.

### Analysis of Quinone Degradation

DCBQ (Sigma-Aldrich) stock solution (10 mM in DMSO) was dissolved in PBS (0.01 M phosphate buffer, 0.0027 M KCl, 0.137 M NaCl, Sigma-Aldrich) to achieve a final concentration of 100 μM. The pH was adjusted by adding NaOH or HCl. Absorbance of DCBQ (Sigma-Aldrich) solutions was measured using an Ocean FX Miniature Spectrometer (Ocean Optics, Ocean-FX-XR1-ES and DH-mini light source) or Thermo Scientific Varioskan LUX. UV–vis spectra were recorded in a closed quartz cuvette (path length = 10 mm) or UV-transparent multiwell plate, focusing on the range of 200–600 nm, as no absorption peaks were observed outside this range. DCBQ concentrations obtained for kinetics analysis were calculated using a calibration curve of standard DCBQ solutions (20–100 μM, λ_max_ = 273 nm). For experiments with ferricyanide or SOD and CAT, the samples were extracted by using ethyl acetate to obtain DCBQ in the organic phase. The reported data are means ± standard errors from three replicates.

Spectroelectrochemical experiments were performed using PocketStat (Ivium Technologies) and an Ocean FX Miniature Spectrometer. Pt wire, platinum mesh, and a saturated Ag/AgCl electrode were used as the working electrode, counter electrode, and reference electrode, respectively. The experiment was done under a nitrogen stream and in dark conditions. A sweep rate of 50 mV s^–1^ and an amplitude of 10 mV were used for sweep voltammetry.

For NMR and LC/MS analysis, 10 mg of DCBQ was dissolved in 5 mL of DMSO and diluted in 100 mL of water. The sample was divided into two: one was covered with aluminum foil and kept in the dark, while the other was subjected to normal laboratory light at room temperature. After 24 h, the samples were freeze-dried (VirTis Benchtop K Freeze-Dryer, SP Industries). Samples were dissolved in 0.75 mL DMSO-*d*_6_, and the NMR spectra were recorded on a Bruker Avance III 500 MHz DCH Cryoprobe spectrometer operating at 500.05 and 125.75 MHz. Heteronuclear single quantum coherence (HSQC) experiments were conducted using standard Bruker methods with the following parameters: number of scans 2, spectral width 13 and 190 ppm, acquisition time 0.14 s (F2) and 5.38 ms (F1). Data were acquired with 1816 and 64 data points for F2 and F1, respectively. All data were processed and analyzed by using Bruker TopSpin 4.1.4. Chemical shifts were calibrated to the central DMSO solvent peak (δ = 2.49 ppm for ^1^H and 39.97 ppm for ^13^C).

For LC/MS analysis, dry samples were dissolved in 1 mL of acetonitrile. The solutions were then diluted to ca. 1 mg mL^–1^ with 20% acetonitrile in water (v/v). DMPO adducts were formed by mixing 1 mM DCBQ and 10 mM DMPO (5,5-dimethyl-1-pyrroline-1-oxide, Fluorochem) in water/acetonitrile (9:1 v/v). Samples were put in LC/MS grade vials with septa and injected into reverse-phased LC with an ACQUITY UPLC-BEH C18 column (2.1 mm × 50 mm, particle size 1.7 μm, Waters Corporation). The sampler and column temperatures were set at 10 and 40 °C, respectively. The mobile phase eluents used were composed of water containing 0.1% formic acid (A) and acetonitrile (B) with a gradient elution program. The elution started with 10% B for 5 min, followed by 100% B for 0.5 min, and was set back to 10% B for 0.5 min. The gradient starts at injection, and the mobile phase flow rate was kept at 0.8 μL min^–1^. MS analysis was performed using a Vion IMS QTof mass spectrometer. All samples were analyzed in positive or negative ion modes using an electrospray ionization source. Data were acquired in a scanning range of 50–1000 *m*/*z* and a scan time of 1 s. MS parameters: capillary voltage = +0.70 kV or −0.70 kV, collision energy = 6 eV (low energy), source temperature = 120 °C, desolvation temperature = 280 °C. Cone gas and desolvation gas flows were set at 50 and 800 L h^–1^, respectively. The acquired data were visualized and analyzed using the Waters UNIFI Scientific Information System or OpenMS. All reported mass numbers have errors less than 5 ppm.

### Electron Paramagnetic Resonance Spectroscopy

Electron paramagnetic resonance (EPR) measurements were completed using a Bruker EMX spectrometer using a Bruker Super High QE (SHQE) cavity resonator (ER 4122 SHQE), Bruker (Germany). The preparation of anaerobic samples for EPR analysis was completed in an MBRAUN UNIlabplus eco glovebox (US). 5,5-dimethyl-1-pyrroline N-oxide (DMPO) was purchased from DOJINDO (Japan). 3-(N-morpholino)­propanesulfonic acid (MOPS), MOPS sodium salt, and Hirschmann microcapillary pipettes (50 μL capacity) were purchased from Sigma-Aldrich (Merck, US). Hirschmann wax seal plates were purchased from Thermo Fisher Scientific (US).

DCBQ stock solutions (10 mM in DMSO) were prepared and subsequently diluted to 1 mM in PBS for measurements, unless specified otherwise. For samples containing DMPO or ferricyanide, 20 mM DMPO or 5 mM potassium ferricyanide, respectively, were added to the buffer solutions prior to the addition of DCBQ. Control samples included PBS pH 7.0, 10% DMSO in PBS pH 7.0, 5 mM potassium ferricyanide in PBS pH 7.0, 1 mM DCBQ in DMSO, 1 mM DCBQ in MOPS pH 7.0, and DCBQ dissolved in HPLC-grade water. Aerobic samples were prepared on the bench, and 50 μL was transferred to the Hirschmann microcapillary pipettes and wax sealed. Anaerobic samples were prepared in the glovebox, and 50 μL was transferred to microcapillary pipettes and wax sealed on both ends.

Samples were placed into a 3 mm outer diameter quartz guiding tube and placed in the EPR resonator for measurement immediately after preparation. EPR measurement parameters: center field = 350.09 mT, field width = 12 mT, microwave power = 2.02 mW, gain = 35 dB, modulation amplitude = 0.1 mT, modulation frequency = 100 kHz, and sweep time = 34.01 s. For control experiments, 5 scans were acquired. For all of the other samples, 20 scans were acquired.

Data was analyzed using MATLAB (version 2021a) with the Easyspin plug-in (version easyspin-6.0.0-dev.54).^50^ Hyperfine couplings were determined from the EPR measurement data and were confirmed by simulation using the Easyspin *garlic* function. To determine radical concentrations, simulations were obtained and scaled to each experimental data set. Double integrals of the simulated spectra were then compared against a calibration curve, obtained from 4-hydroxy 2,2,6,6-tetramethylpiperidine 1-oxyl (TEMPOL) samples at different concentrations, and measured under the same conditions as the samples of interest.

### Square Wave Voltammetry

Square wave voltammetry was carried out in an electrochemical cell with FTO-coated glass as the working electrode, platinum mesh as the counter electrode, and Ag/AgCl (saturated KCl) as the reference electrode using EmStat3 Blue. Carbonate-buffered saline solutions with pH 7.0 or 9.0 were used as the electrolyte. The concentrations of DCBQ and ferricyanide used were 500 μM and 2.5 mM, respectively. The first measurement was taken directly after addition of DCBQ and ferricyanide, and subsequent measurements were taken every 5 min thereafter. SWV measurement parameters: equilibrium time = 5 s, E_begin_ = 0.5 V, E_end_ = -0.7 V, E_step_ = 0.015 V, amplitude = 0.05 V, frequency = 30.0 Hz, E_standby_ post measurement = 0.5 V for 30 s. The background current was obtained when only the electrolyte was present, and all curves with DCBQ or DCBQ and ferricyanide were subtracted from the background current. Three technical replicates were performed for each condition.

For each replicate, all visible peaks in the voltammograms were identified, and their peak values were extracted at each time point. For time points after which the peak had disappeared, the current value used was that at the same potential as the peak before it had disappeared. The three replicates were then averaged, and their standard deviations were taken as experimental errors.

The data were fit to a set of differential equations using a custom Python script, optimizing the parameters of the differential equations for a minimal difference between the fit and the data. The experimental error was used directly to estimate the covariance of the fitted parameters.

### Cell Culture

Cell cultures of wild-type *Synechocystis* sp. PCC6803 and BG11 were prepared according to a previously reported recipe.^51^ The cell cultures were grown under photoautotrophic conditions, exposed to continuous white light at an intensity of 1 mW cm^–2^ and maintained at a temperature of 30 °C with shaking at 100 rpm. The pH of the BG11 medium used for cell cultures was adjusted to 7.8 and supplemented with NaHCO_3_.

The growth of the cell cultures was assessed by measuring the optical density at 750 nm (OD_750_) using an Agilent Cary 60 UV–vis spectrophotometer. The concentration of chlorophyll *a* was determined based on a previously reported method.^52^

### Oxygen Evolution Measurement

Oxygen evolution assays were performed using a Clarke electrode consisting of an S1 Oxygen Electrode Disc, Oxylab control unit, LH36/2R light housing, and DW2/2 electrode chamber (Hansatech Instruments). Suspensions of *Synechocystis* (1.5 mL, 10 nmol Chl *a* mL^–1^) were measured in BG11 pH 7.0 with or without addition of 200 μM benzoquinones and 1 mM potassium ferricyanide (Sigma-Aldrich). The oxygen level was measured at 25 °C with 1 min dark condition, followed by two cycles of alternating light and dark conditions for 90 and 120 s, respectively. The samples were irradiated with 750 μmol photons m^–2^ s^–1^ of red light centered at 650 nm. The net photosynthetic oxygen evolution rates were obtained by subtracting the rate of oxygen production under light conditions from the rate of oxygen uptake under dark conditions. The reported data are means ± standard errors from three biological replicates.

### Biophotoelectrochemical Systems

IO ITO electrodes were prepared by using a template infiltration method. FTO-coated glass (8 Ω sq^–1^, Sigma-Aldrich) was cleaned by sonication in isopropanol and ethanol at 37 kHz for 15 min and then dried at 150 °C. A circular hole with a diameter of 1 cm was punched into Scotch Magic tape (width of 19 mm, 3M) to create a mold with a defined geometric surface area, which was then attached to the conductive side of FTO-coated glass. The glass with molds was treated with UV-ozone for 15 min. Polystyrene microbeads (20 μm, 10% w/v aqueous suspension, Supelco) were centrifuged at 10,000 rpm for 8 min, and 40% (v/v) of the supernatant was removed. Methanol (Thermo Scientific) was added to the remaining supernatant (25% (v/v) of methanol in the final mixture), and the polystyrene microbeads were redispersed by vortexing the mixture. The dispersion (30 μL) was drop cast into the mold, allowed to settle, and self-assembled at a temperature of 5 °C for 16 h. The glass piece was then sintered at 100 °C for 12 min to form a tightly packed polystyrene template.

ITO nanoparticles (80 mg, <50 nm diameter, Thermo Scientific) were sonicated in 300 μL of MeOH/water mixture (5:1 v/v) at 37 kHz for 5 h while keeping the temperature below 20 °C. The polystyrene template was then drop cast with 9.5 μL of ITO dispersion and immediately covered by a plastic Petri dish to avoid quick evaporation. The electrodes were put in a cold room for 30 min and then moved to room temperature until dry. Then, the electrodes were heated in a furnace (Carbolite furnace, ELF) at a ramp rate of 1 °C min^–1^ from room temperature to 500 °C, held at 500 °C for 20 min, and then cooled back to room temperature.

Concentrated cell suspension (250 μL of 150 nmol Chl *a* mL^–1^) prepared from cell culture (OD_750_ = 1) was loaded onto the IO ITO electrode and incubated for 18 h under dark conditions to allow biofilm formation. The electrode was rinsed to remove excess unattached cells. Chronoamperometry experiments were carried out with a three-electrode setup consisting of a cell-loaded IO ITO as the working electrode, Ag/AgCl (sat. KCl) as the reference electrode, and platinum mesh (nominal aperture 0.12 mm, wire diameter 0.04 mm, Goodfellow) as the counter electrode. DBCQ and potassium ferricyanide were added into the electrolyte (PBS pH 7.0), resulting in a concentration of 200 μM and 1 mM, respectively. The system was kept in the dark for 10 min until the background current stabilized. The working electrode was irradiated with a collimated red LED (λ = 680 nm, 1 mW cm^–2^ s^–1^, ThorLabs) for 24 h with 3 min (no mediator) or 5 min (with mediators) light off in each hour to establish the baseline unless specified otherwise. All measurements were performed using an Ivium Technologies CompactStat. The reported data are means ± standard errors from three biological replicates.

Post photoelectrochemical measurement, the cell-loaded electrode was scraped and suspended into 500 μL of methanol. The suspension was then sonicated at 37 kHz for 1 h in ice water and centrifuged at 10,000 rpm for 5 min. The supernatant was analyzed using UV–vis spectrophotometry for chlorophyll *a* quantification (ε_665 nm_ = 79.95 (mg Chl *a*)^−1^ mL cm^–1^).^52^

## Supplementary Material



## Notes

Supplementary Figures S1–S21; Supplementary notes: Square wave voltammetry optimization, kinetic modeling, related figures (Figures SN1–SN5), and tables (Tables SN1–SN2) (PDF)

## Abbreviations

CAT: catalase
CHQ–OH: 2-chloro-6-hydroxy-1,4-hydroquinone
DCBQ: 2,6-dichloro-1,4-benzoquinone
DCHQ: 2,6-dichloro-1,4-hydroquinone
DCSQ: 2,6-dichloro-1,4-semiquinone
DCSQ_d_: derivatives of 2,6-dichloro-1,4-semiquinone
DMPO: 5,5-dimethyl-1-pyrroline N-oxide
DMSO: dimethyl sulfoxide
EPR: electron paramagnetic resonance
FeCN: ferricyanide
FTO: fluorine-doped tin oxide
LC-MS: liquid chromatography–mass spectrometry
NMR: nuclear magnetic resonance
OEC: oxygen-evolving complex
PSII: photosystem II
PBS: phosphate-buffered saline
PQ: plastoquinone
PQH_2_: plastoquinol
Q_A_/Q_B_: primary/secondary quinone electron acceptors in PSII
ROS: reactive oxygen species
SHE: standard hydrogen electrode
SN: supplementary notes
SOD: superoxide dismutase
SWV: square wave voltammetry
