# Supplementary material for: Unraveling Quinone Degradation Enables Stabilization Using Redox Helpers in Biological and Electrochemical Systems
Source: J Am Chem Soc. 2026 Apr 9;148(15):15812–25. doi: 10.1021/jacs.5c22307 (PMC13107435; doi:10.1021/jacs.5c22307)
Supplement: Supplementary file 1 [file ja5c22307_si_001.pdf]

SUPPORTING INFORMATION

UNRAVELLING QUINONE DEGRADATION ENABLES STABILIZATION USING REDOX HELPERS IN BIOLOGICAL AND ELECTROCHEMICAL SYSTEMS

Shella J. Willyam, [1] *Robin A. Scullion,* [1] *Sarah F. Chapman,* [2] *Eleanor R. Clifford,*[2] *Maxie M. Roessler,* [2] *Jenny Z. Zhang*[1]*

[1]Yusuf Hamied Department of Chemistry, University of Cambridge, Lensfield Road, Cambridge, CB2 1EW, United Kingdom

[2]Department of Chemistry and Centre for Pulse EPR Spectroscopy, Imperial College London, Molecular Sciences Research Hub, 82 Wood Lane, London, W12 0BZ, United Kingdom

* Email: jz366@cam.ac.uk

This document contains:

Supplementary Figures S1-S21

Supplementary Notes (including Figures SN1-SN5 and Table SN1-2)

SUPPLEMENTARY FIGURES

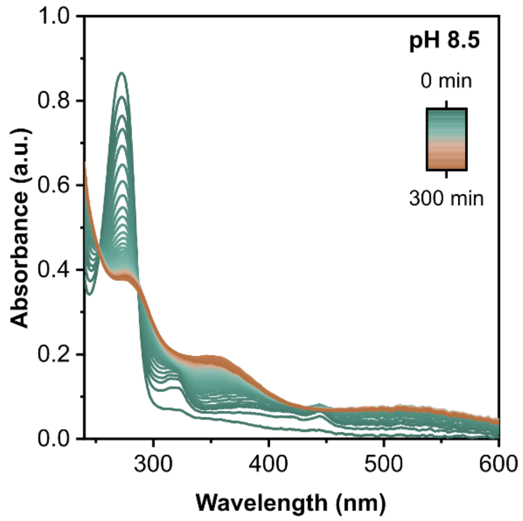

Figure S1. Representative UV-vis spectra of DCBQ in PBS (pH 8.5) over 300 minutes at room temperature.

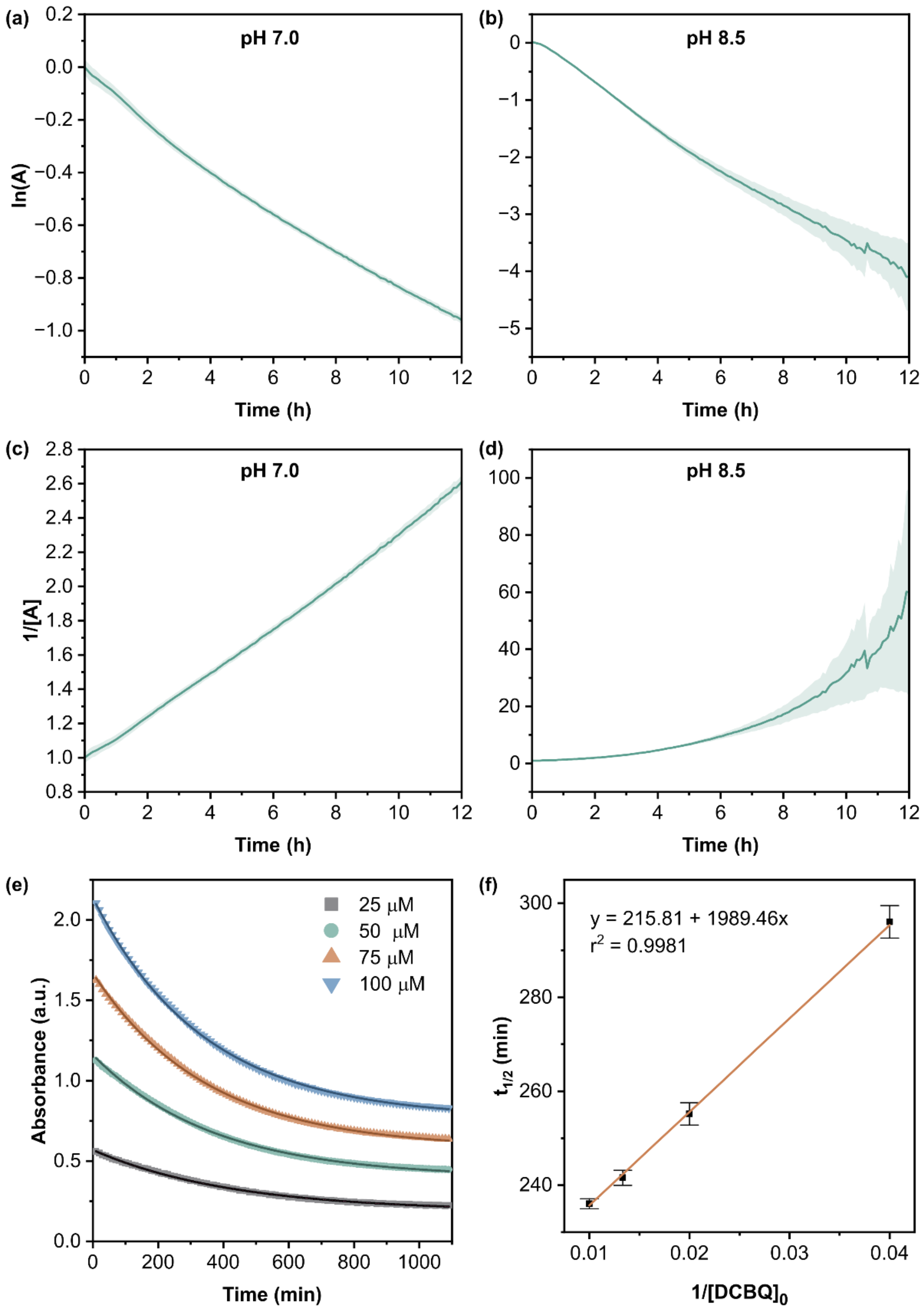

Figure S2. (a–b) Natural logarithm of DCBQ absorbance plotted against time in PBS at pH 7.0 and 8.5, respectively, to assess first-order kinetics. **(c–d)** Plots of the inverse DCBQ absorbance versus time in PBS at pH 7.0 and 8.5, respectively, to evaluate second-order kinetics. Because DCBQ degradation at pH 7.0 shows comparable linearity for both first- and second-order models, **(e)** time-dependent degradation profiles were examined across different initial DCBQ concentrations. **(f)** Corresponding plot of half-life as a function of the inverse initial DCBQ concentration.

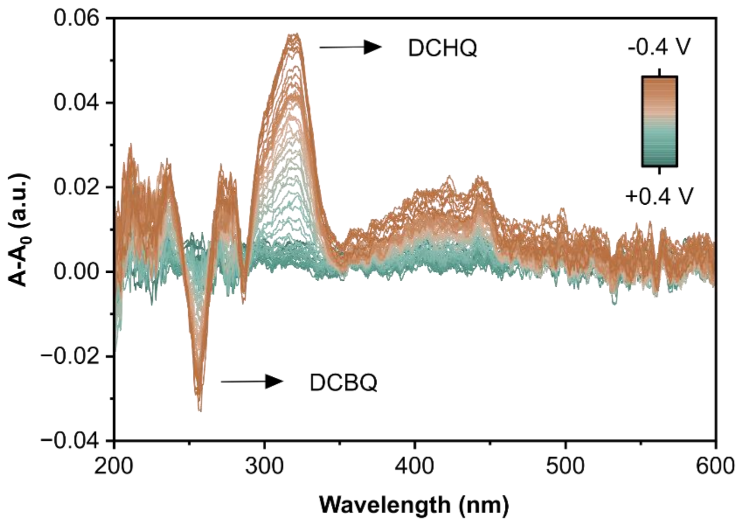

Figure S3. Representative curve of change in DCBQ absorbance in PBS (pH 7.0) during linear sweep voltammetry at room temperature. Scan rate = 50 mV/s.

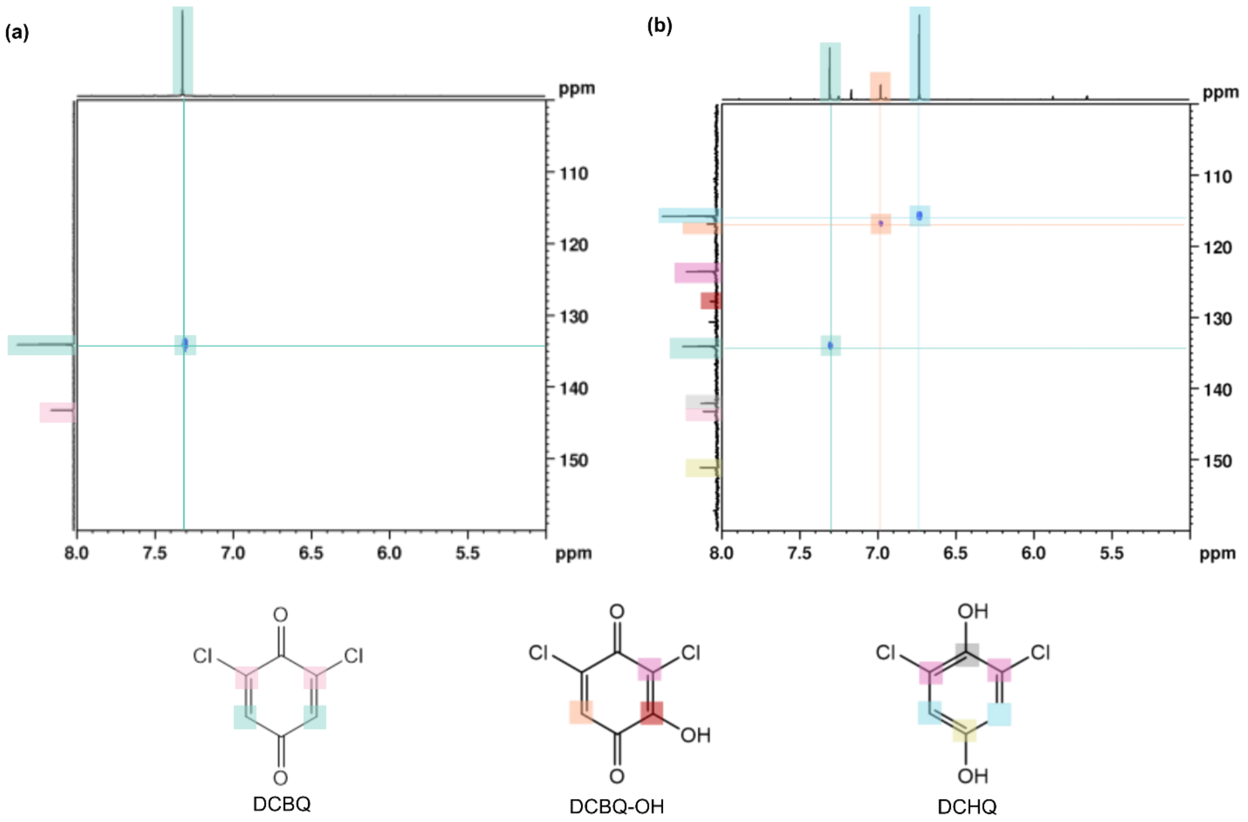

Figure S4. ^{1}H-^{13}C HSQC spectra of DCBQ **(a)** right after dissolution in DMSO-d_6, **(b)** after 24 h in deionized water under illumination of ambient laboratory light. Sample (b) was freeze-dried and redissolved in DMSO-d_6.

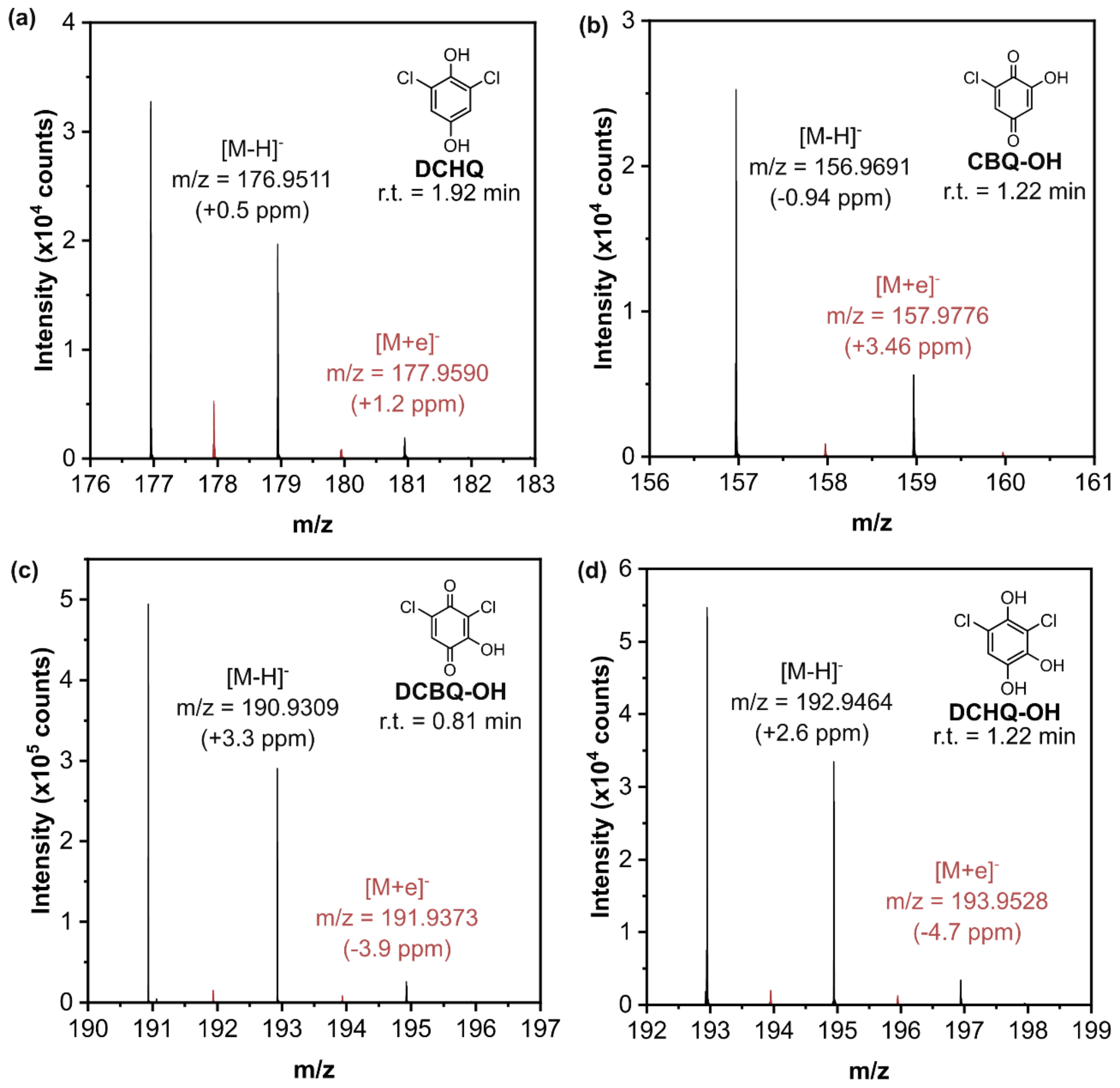

Figure S5. ESI(-) mass spectra of identified major products of DCBQ degradation after 24 h in HPLC-grade water under dark condition.

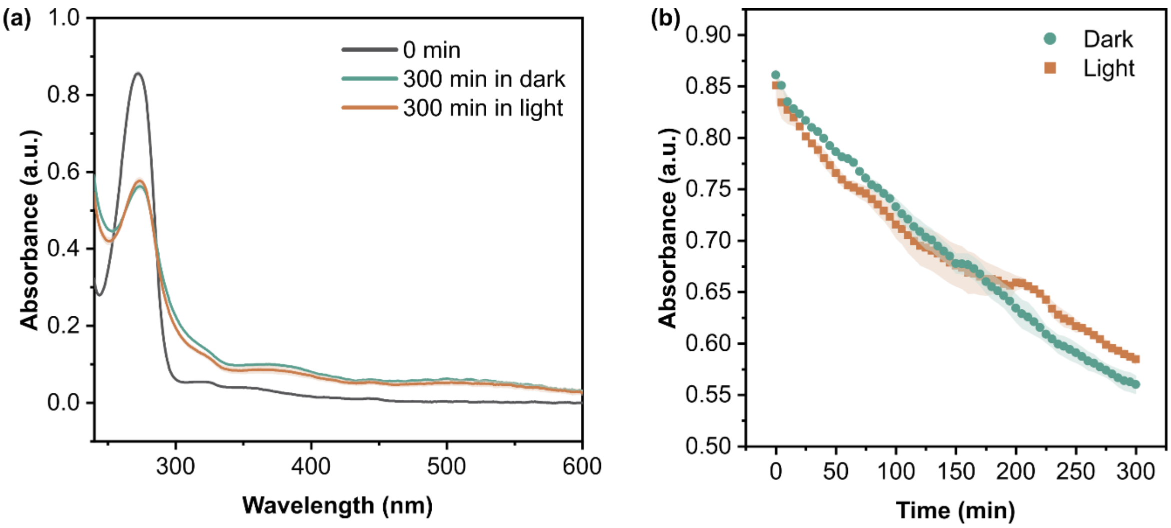

Figure S6. (a) UV-vis spectra of DCBQ in PBS (pH 7.0) over 300 minutes in dark and light conditions at room temperature. **(b)** Comparison of DCBQ decay kinetics under dark and light conditions. Light condition: white LED, 5 mW cm^{-2}. Data are reported as mean ± standard error (n = 3).

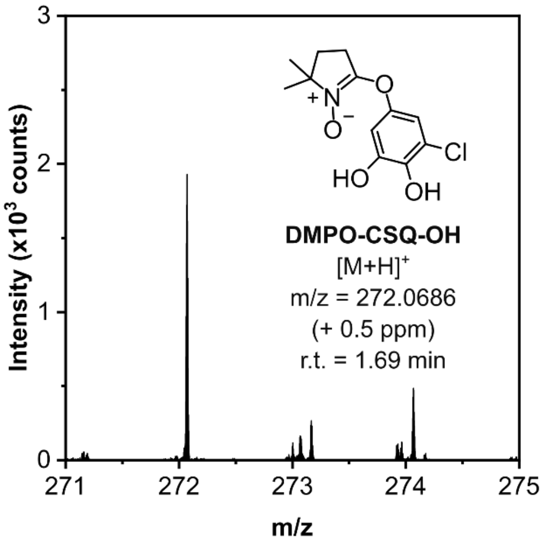

Figure S7. ESI(-) mass spectra of identified DMPO-adduct of semiquinone intermediate during DCBQ degradation after 24 h in HPLC-grade water under dark condition.

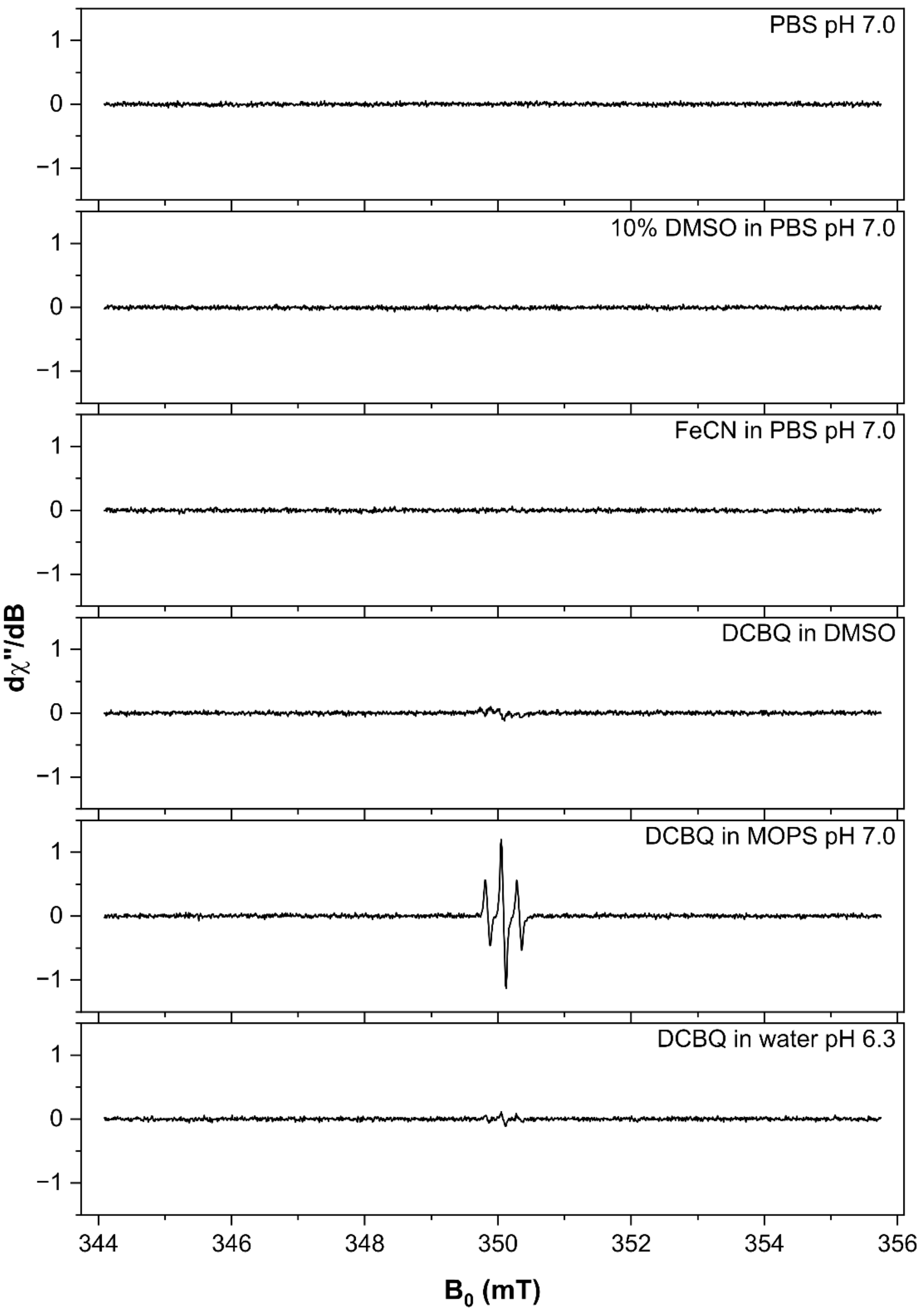

Figure S8. EPR spectra of control experiments. The spectra were recorded at 9.849 GHz and room temperature.

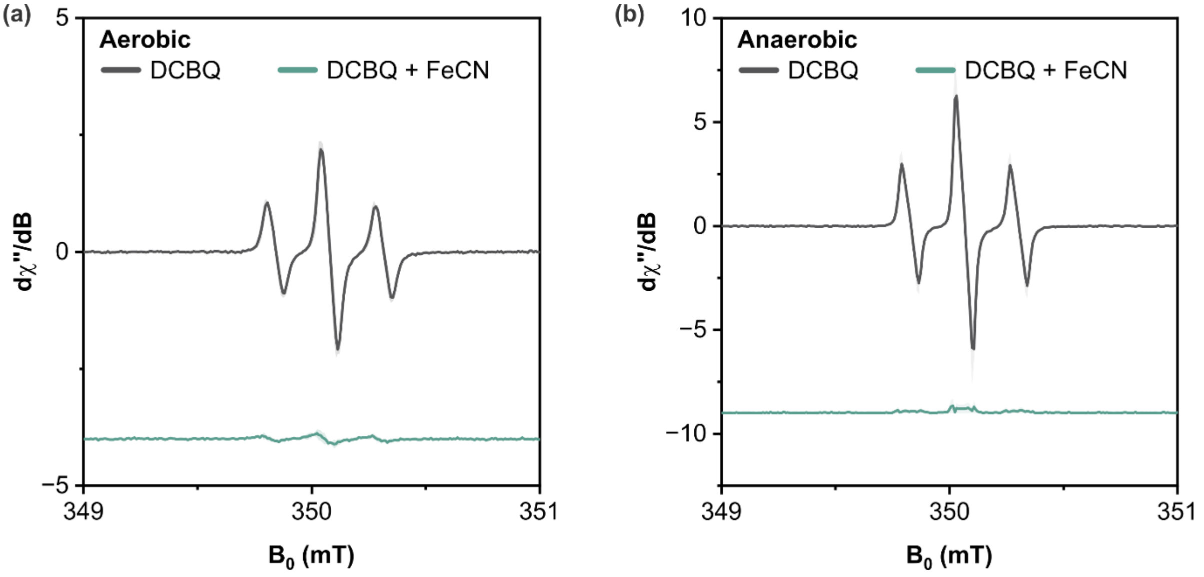

Figure S9. EPR spectra of DCBQ solutions (in PBS pH 7.0) in the absence and presence of ferricyanide (FeCN) under **(a)** aerobic and **(b)** anaerobic conditions. The spectra were recorded at 9.849 GHz and room temperature.

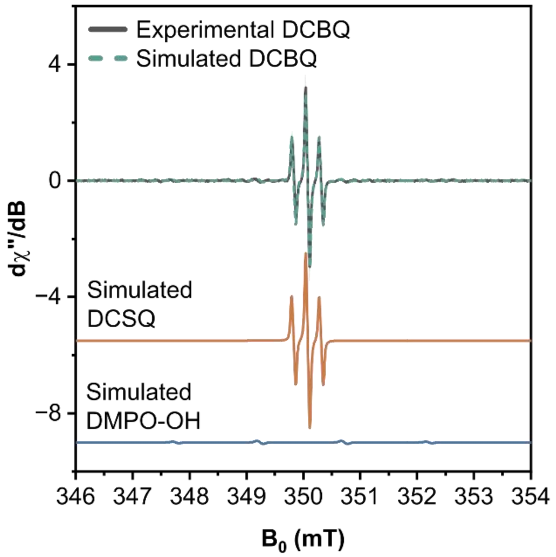

Figure S10. EPR spectra of DCBQ solution (in PBS pH 7.0) in the presence of DMPO under anaerobic conditions, along with the simulated spectra of DCSQ (g = 2.0103, A_{H1} = 2.4 mT, A_{H2} = 2.4 mT), DMPO-OH (g = 2.0106, A_N = A_H = 4.18 mT), and both combined. The spectra were recorded at 9.849 GHz and room temperature.

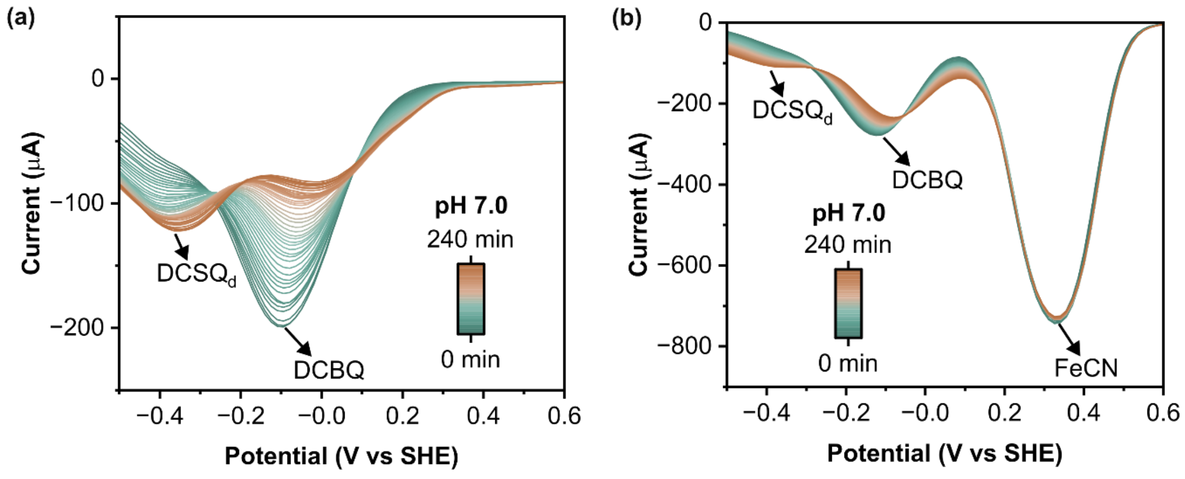

Figure S11. Square wave voltammograms of DCBQ in carbonate buffer at pH 7.0 **(a)** without and **(b)** with ferricyanide.

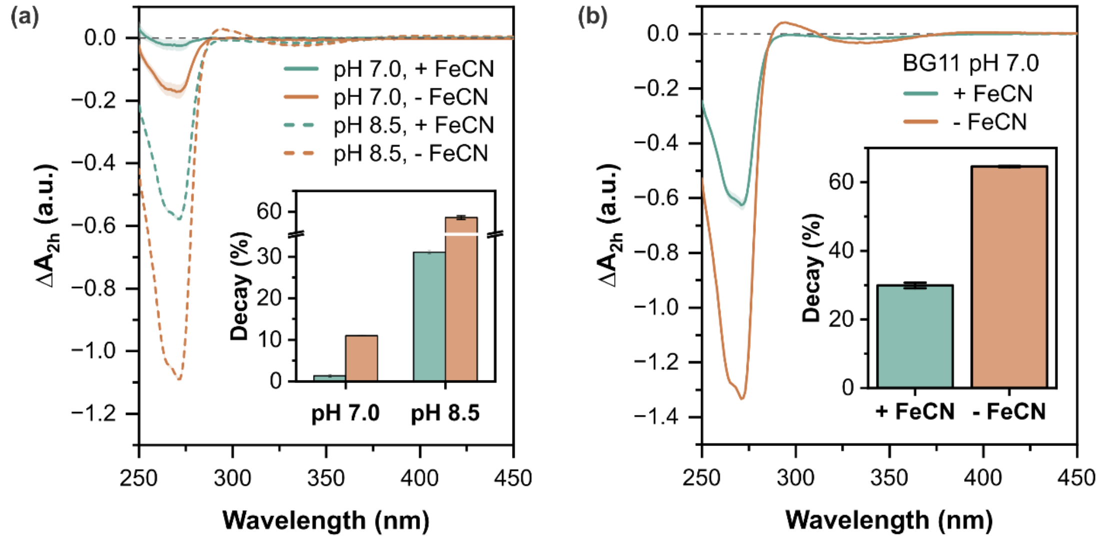

Figure S12. Change in absorption spectra of DCBQ in ethyl acetate extract after 2 hours of dissolution in **(a)** PBS pH 7.0 and 8.5 or **(b)** BG11 media with and without the addition of ferricyanide (FeCN). The characteristic absorbance peak of DCBQ is at around 275 nm.

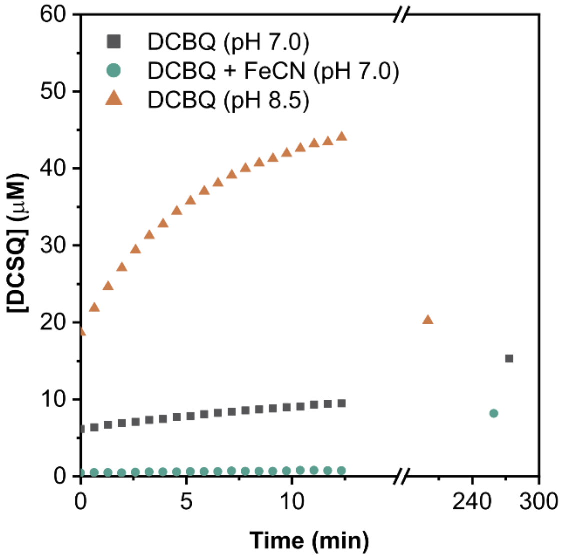

Figure S13. Temporal concentrations of $DCSQ_d$ radicals obtained from EPR spectra of DCBQ solutions in PBS under aerobic conditions.

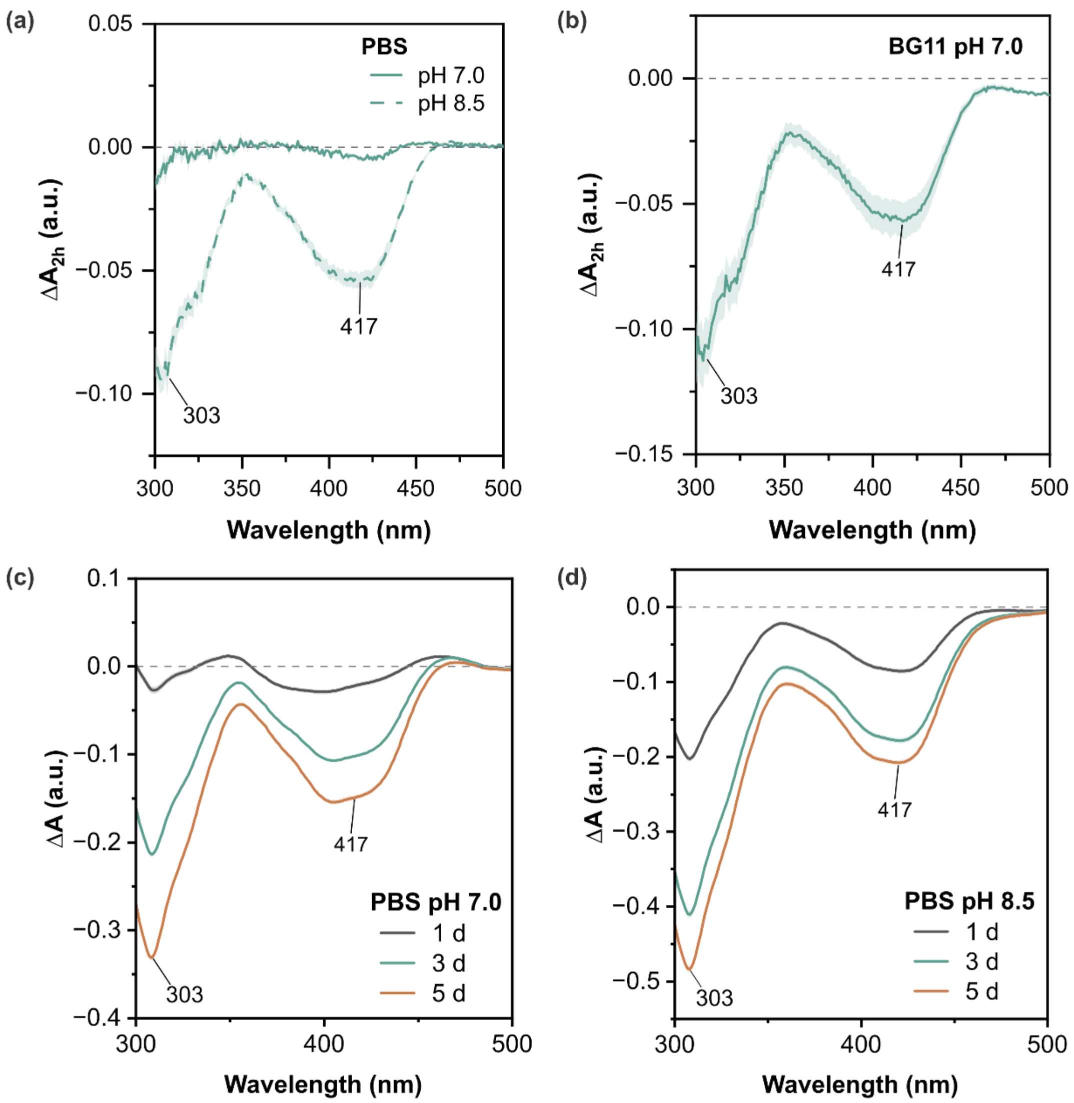

Figure S14. Changes in the absorption spectra of ferricyanide following 2 hours of dissolution in **(a)** PBS pH 7.0 and 8.5, and **(b)** BG11 pH 7.0, as well as during long-term dissolution in **(c)** PBS pH 7.0 and **(d)** pH 8.5 at room temperature. The characteristic ferricyanide absorbance peaks are at around 303 and 417 nm. Data are reported as mean ± standard error (n = 3).

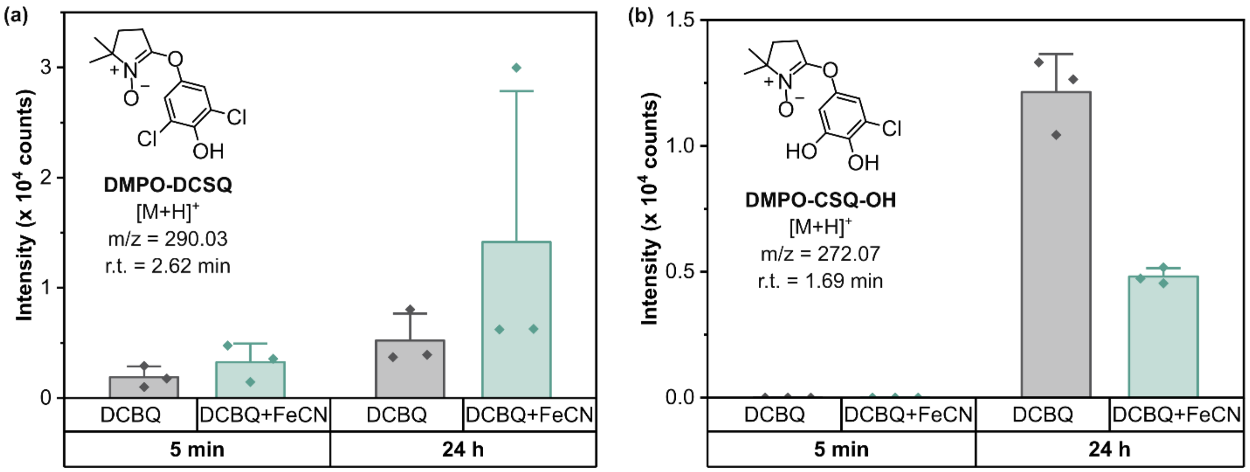

Figure S15. **(a)** DMPO-DCSQ **(b)** DMPO-CSQ-OH adducts abundance in DCBQ solution with and without ferricyanide after certain timepoints. CSQ-OH is the intermediate of one of the major DCBQ degradation products.

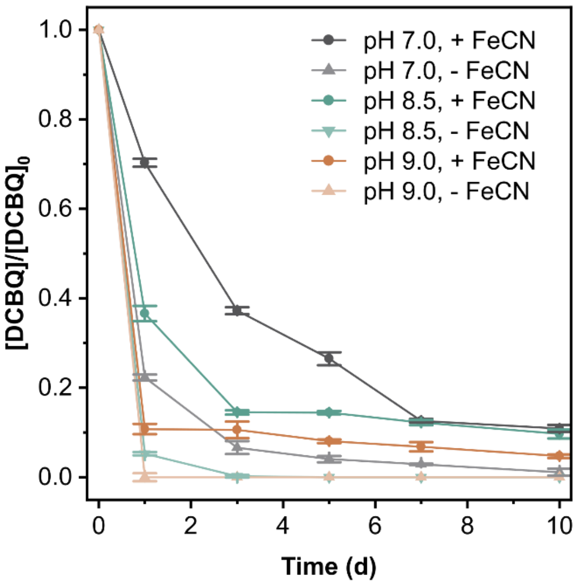

Figure S16. Residual amount of DCBQ in PBS pH 7.0 and 8.5, and carbonate-buffered saline pH 9.0 after 1 to 10 days in the absence and presence of FeCN. Data are reported as mean ± standard deviation (n = 3).

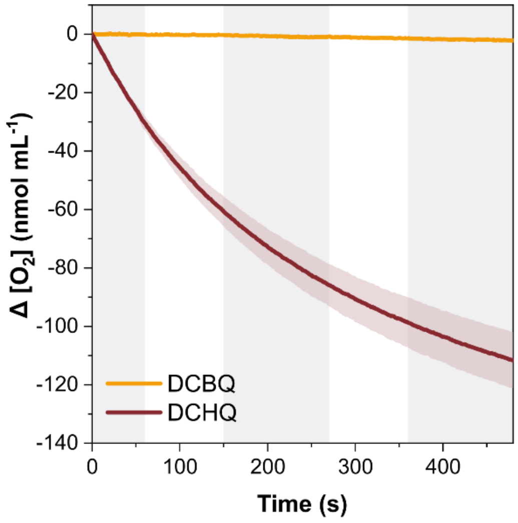

Figure S17. Temporal changes in oxygen concentration in DCBQ and DCHQ solutions (in PBS pH 7.0 at 25ºC). Grey and white shades indicate dark and light periods, respectively. Light conditions: 680 nm, 10 mW cm^{-2}. Data are reported as mean ± standard error (n = 3).

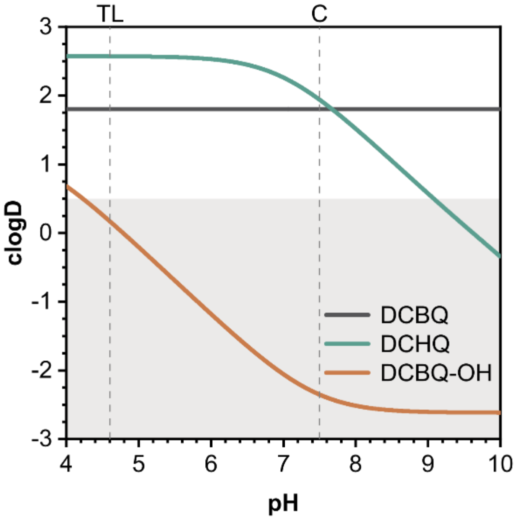

Figure S18. Predicted membrane permeability of DCBQ in oxidized, reduced, and hydroxylated forms at different pH values. LogD values were calculated in ChemAxon Marvin. White area indicates suitable predicted lipophilicity to penetrate lipid membranes. TL and C denote the pH of the thylakoid lumen and cytoplasm, respectively.[37]

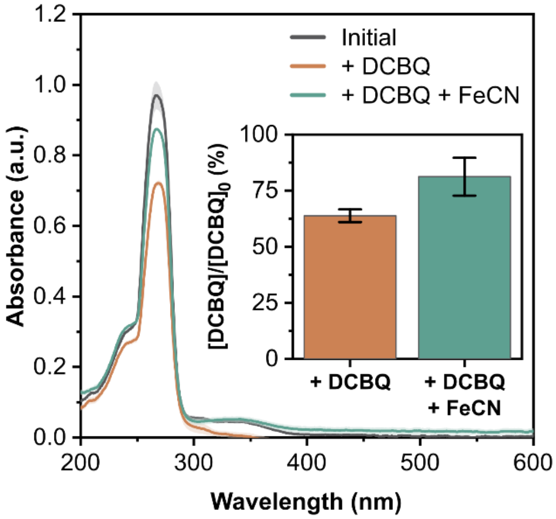

Figure S19. Quantification of the remaining DCBQ in PBS (pH 7.0) amount after 2 hours of photoelectrochemical experiment at 25 ºC. Light conditions: 680 nm, 1 mW cm^{-2}, cycle of 55 min on and 5 min off. Data are reported as mean ± standard error (n = 3).

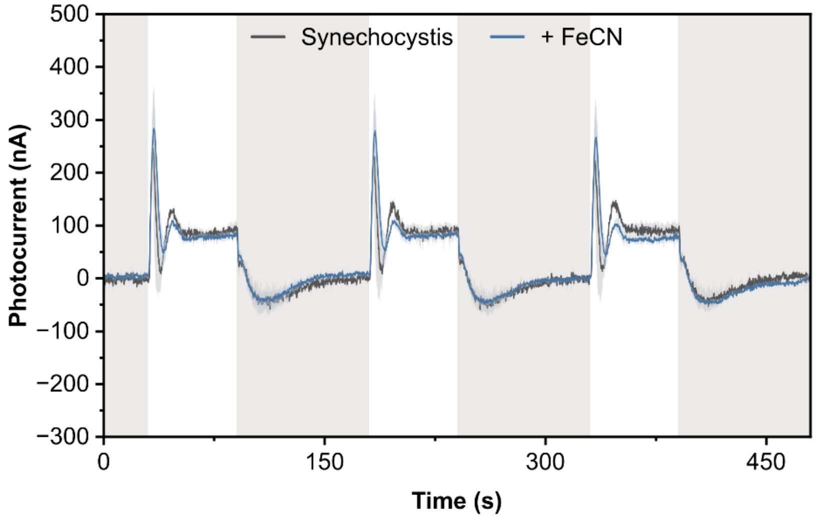

Figure S20. Photocurrent profiles of Synechocystis biofilm in the absence and presence of 1 mM ferricyanide (FeCN) at 25 ºC. White and grey shades indicate light and dark conditions, respectively. Light conditions: 680 nm, 1 mW cm^{-2}, cycle of 60 s on and 90 s off. Applied potential = +0.4 V vs Ag/AgCl (sat. KCl). Electrolyte: phosphate-buffered saline pH 7.0. Data are reported as mean ± standard error of three biological replicates.

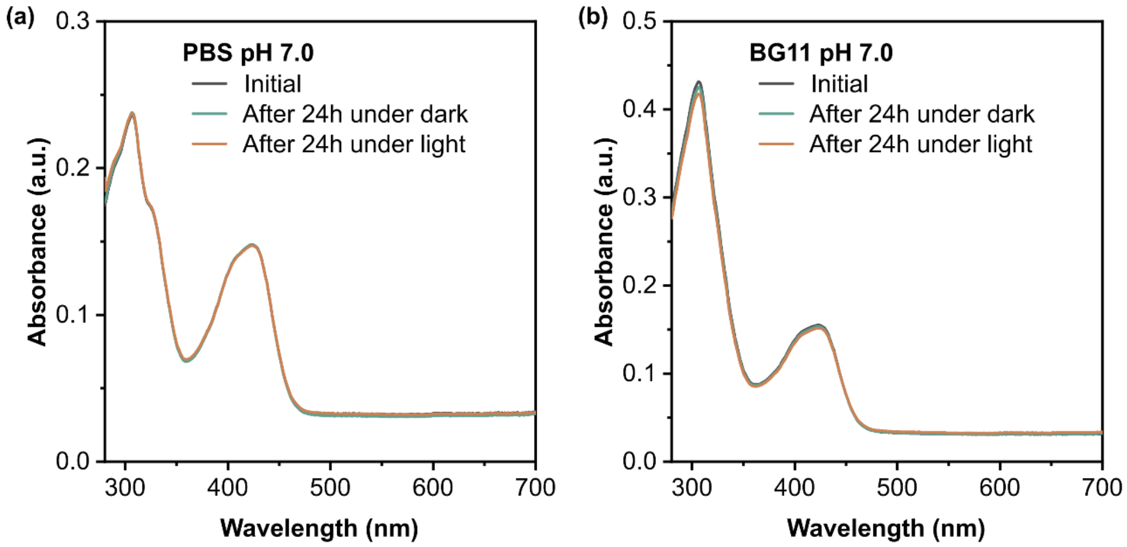

Figure S21. UV–vis spectra of ferricyanide before and after 24 h under dark and light conditions in (a) PBS at pH 7.0 and (b) BG11 at pH 7.0. Light condition: 680 nm, 1 mW cm^{-2}. Data are reported as mean ± standard error (n = 3).

SUPPLEMENTARY NOTES

Modelling of DCBQ degradation from square-wave voltammetry

SWV peak as a proxy of DCBQ concentration

In square-wave voltammetry (SWV), the peak height reflects the differential current (Δi_p), which is related to the concentration of an electroactive species, O, according to the equation:

$$\Delta i_p = \frac{nFAD_O^{\frac{1}{2}}C_O}{\pi^{\frac{1}{2}}\, t_p^{\frac{1}{2}}}\Delta\psi_p$$

where n is the number of electrons transferred, F is Faraday's constant, A is the electrode area, D_O is the diffusion coefficient of the species O, t_p is the experimental timescale, and $\Delta\psi_p$ is a dimensionless parameter characterizing the peak current. Under conditions of constant temperature and a fixed timescale across replicates, Δi_p is directly proportional to the concentration C_O.

SWV measures the differential current ($\Delta i_p = i_{forward} - i_{reverse}$), effectively suppressing the broad diffusion-limited currents from species reduced at more positive potentials. Because SWV operates on a rapid timescale determined by its pulse frequency, the technique is highly sensitive to electron transfer kinetics. This kinetic dependence allows SWV to resolve the signals of compounds with similar midpoint potentials but different heterogeneous rate constants, such as DCBQ and ferricyanide. The brief millisecond pulses mean the slower-reacting species (DCBQ) requires a larger overpotential to drive the reaction, shifting its reduction peak negatively and separating it from the faster-reacting ferricyanide. This also explains why the quasi-reversible reduction of DCBQ appears at a more negative potential in SWV than in a continuous, slower cyclic voltammetry (CV) sweep. Consequently, by optimizing the frequency and pulse amplitude, the mass-transfer limited current from ferricyanide appeared as a stable, featureless baseline rather than an overlapping signal.

Optimizing square-wave voltammetry (SWV) method

The SWV analysis protocol was established following considerable optimization. The working electrode material, FTO-coated glass, was chosen over others (e.g., glassy carbon) since it has favourable electron transfer kinetics with ferricyanide that are significantly faster than for DCBQ. This kinetic difference results in a clear separation of their respective reduction peaks, which was essential for quantification of each component. Following this, other SWV parameters (e.g., pulse amplitude, frequency, potential range) were systematically adjusted to maximize signal-to-noise ratio and ensure clear peak resolution and separation. The 5-minute interval and brief re-oxidation after each measurement were used to capture the degradation kinetics with sufficient temporal resolution without introducing

experimental drift. Furthermore, it was necessary to replace phosphate-buffered saline with carbonate-buffered saline because phosphate ions are known to form passivation layers on the electrode surface, a process which can interfere with the analysis and lead to inaccurate analytical measurements.

A ferricyanide concentration of 2.5 mM (with 0.5 mM DCBQ) was selected to ensure high signal-to-noise ratios and robust peak resolution for kinetic fitting. Importantly, this maintains the same 5:1 molar ratio of helper-to-mediator used in the oxygen evolution and biophotoelectrochemical measurements (1 mM FeCN : 0.2 mM DCBQ), ensuring that the kinetic stabilization mechanism remains comparable across both experimental scales while accommodating the sensitivity requirements of the electrochemical technique.

Validation of SWV signal integrity

A key consideration for this analysis is the observation that the ferricyanide peak current clearly decreases over time (Figure 3b and Figure S11). This is expected as ferricyanide is consumed while re-oxidizing the semiquinones and potentially reactive oxygen species.

It is therefore necessary to confirm that this changing ferricyanide concentration does not interfere with the measurement of the DCBQ peak at lower potentials. Control experiments were conducted using only ferricyanide in the electrolyte (Figure SN1). These controls demonstrated that even as the ferricyanide peak changes, it does not significantly affect the background current in the potential region where the DCBQ reduction peak is observed (approximately 0 to -0.2 V vs SHE). Therefore, the DCBQ peak current can be reliably used as a proxy for its concentration, independent of the changes in the ferricyanide signal, even though the ferricyanide itself is consumed over the course of experiment.

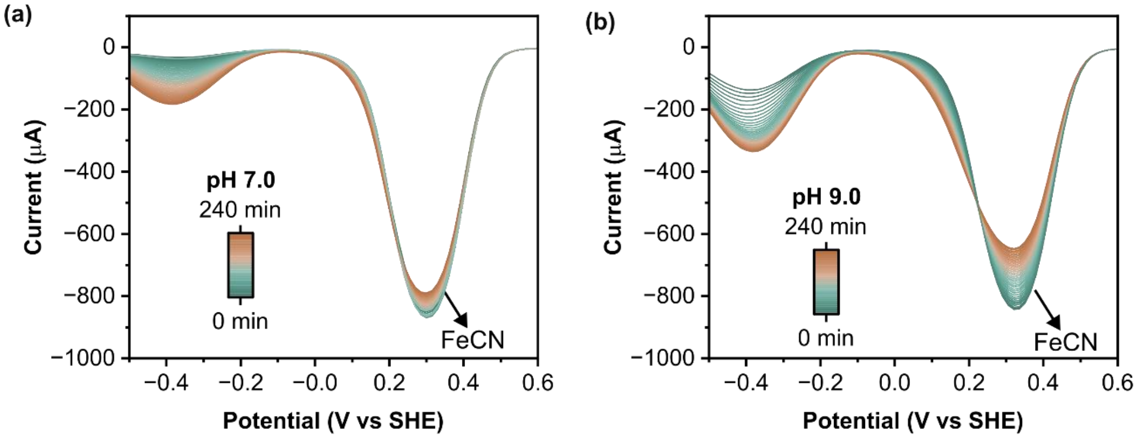

Figure SN1. SWV of ferricyanide at **(a)** pH 7.0 and **(b)** pH 9.0.

Does DCBQ degradation fit exponential decay?

We first fitted a simple exponential decay model (indicative of a first-order reaction) to describe the degradation of DCBQ with and without ferricyanide at pH 7.0 and pH 9.0. The fitted parameters, along with their errors, are detailed in Table SN1, while the fit plots are shown in Figure SN2.

$$[DCBQ] = A + Be^{-kt}$$

The goodness of fit was evaluated by analyzing the residuals (i.e., the differences between each experimental value and the model prediction). A satisfactory model should yield residuals that are relatively low, approximately normally distributed around zero, and exhibit minimal systematic trends over time. At pH 7.0, both for DCBQ alone and for DCBQ with ferricyanide, the residuals were mostly less than 1 and symmetrically distributed around zero, although the ferricyanide data exhibited a slightly bimodal distribution (Figure SN3). In contrast, at pH 9.0 the residuals were substantially larger (reaching up to 10 when ferricyanide was present) and displayed a clear temporal trend. The histogram of the residuals at this pH also revealed a skewed distribution, with a bias toward negative values. These findings, particularly for the DCBQ plus ferricyanide system at pH 9.0, suggest that the exponential decay model does not adequately capture the degradation kinetics under these conditions.

Table SN1. Obtained parameters from fitting of SWV data to a simple exponential decay. Parameters A and B represent the peak current and are given in arbitrary units as the current is used as a relative proxy for concentration.

pH	Condition	k	A	B
7.0	DCBQ	$(1.20 \pm 0.18) \times 10^{-2}$	67.4 ± 8.3	169 ± 7
7.0	DCBQ + FeCN	$(4.34 \pm 1.37) \times 10^{-3}$	199 ± 17	89.2 ± 14.8
9.0	DCBQ	$(6.82 \pm 0.60) \times 10^{-2}$	42.8 ± 0.5	143 ± 11
9.0	DCBQ + FeCN	$(3.30 \pm 0.25) \times 10^{-2}$	71.0 ± 1.6	213 ± 11

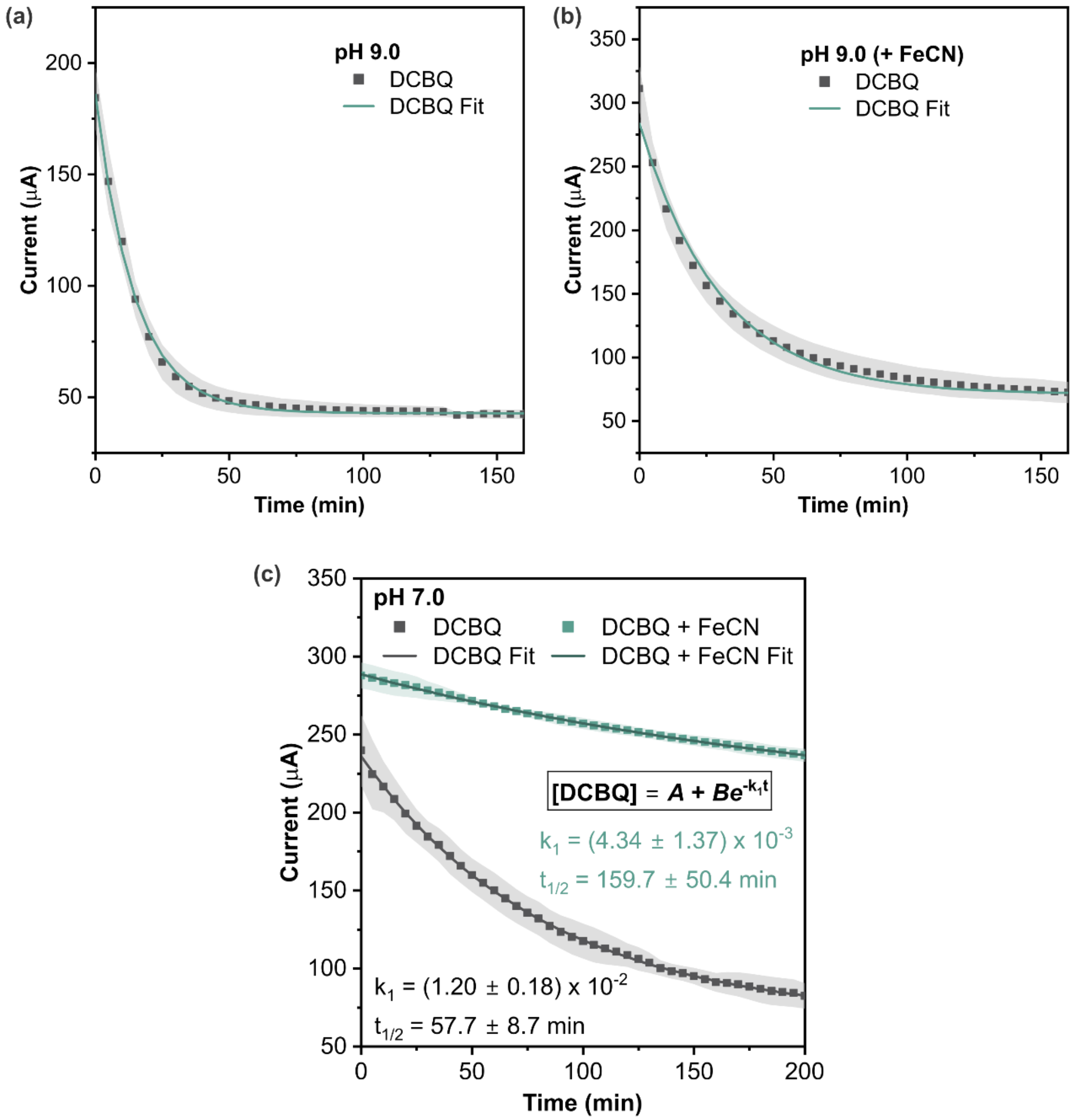

Figure SN2. Plots of DCBQ peak current over time at pH 9.0 **(a)** without and **(b)** with ferricyanide, and **(c)** at pH 7.0 along with their simple exponential fittings.

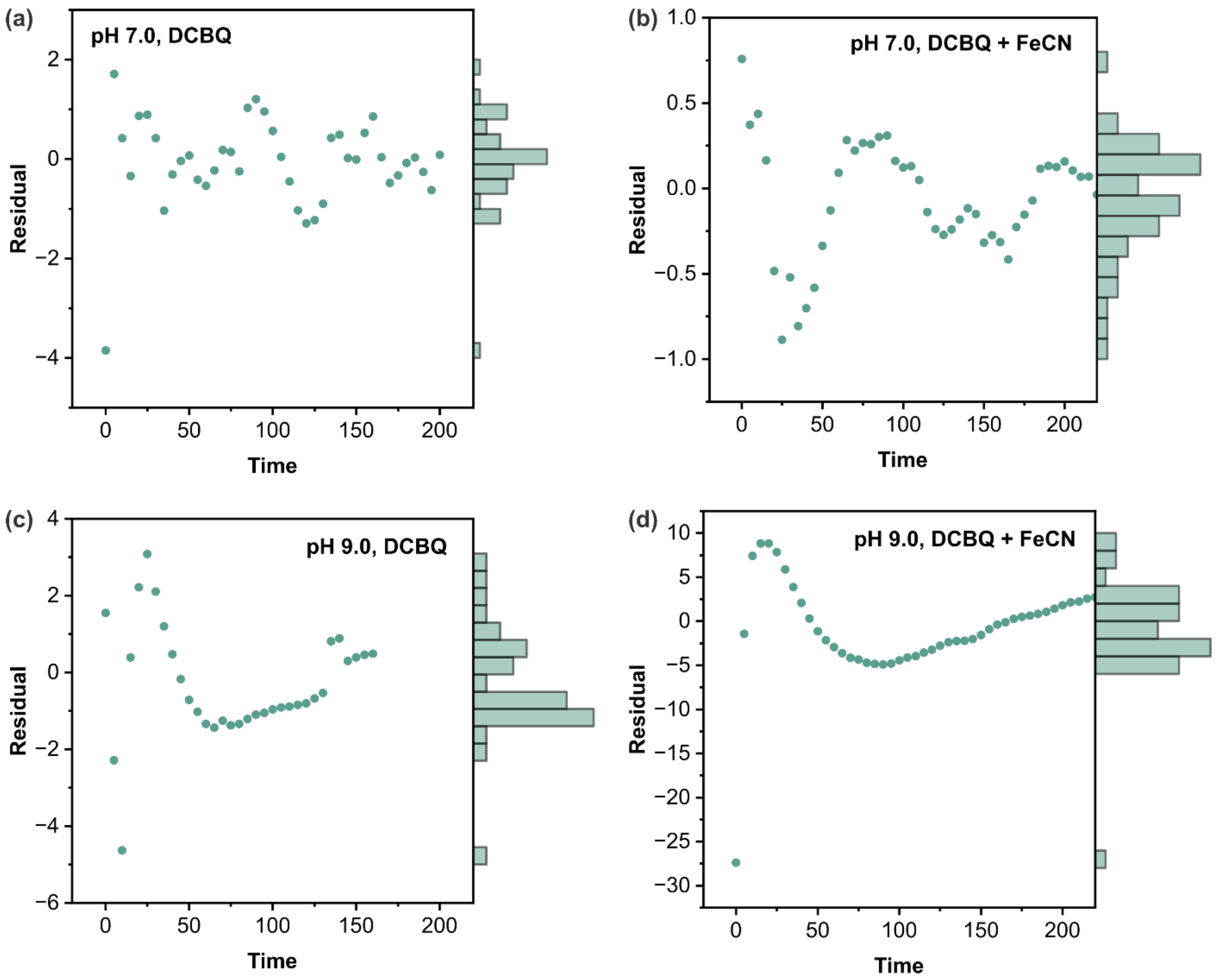

Figure SN3. Residual plots of simple exponential fittings (plots of the fittings are shown in Figures 4c and SN2).

A differential equation model for DCBQ degradation

In light of these observations, we extended our analysis by considering a more complex model. We propose that the degradation of DCBQ follows the scheme:

$$DCBQ \rightleftharpoons DCSQ_d \rightleftharpoons sink$$

which is modelled by the following set of coupled differential equations:

$$\frac{d[DCBQ]}{dt} = -k_1[DCBQ] + k_{-1}[DCSQ_d]$$

$$\frac{d[DCSQ_d]}{dt} = -k_{-1}[DCSQ_d] + k_1[DCBQ] - k_2[DCSQ_d] + k_{-2}[sink]$$

$$\frac{d[sink]}{dt} = k_2[DCSQ_d] - k_{-2}[sink]$$

This model is a simplified version of Scheme 1, and assumes that the first two reaction steps can be condensed into a single pseudo-first-order (in DCBQ) reaction. As discussed in the main text, the reaction order of these initial steps of DCBQ degradation was found to be highly dependent on pH. To check that our assumption here still holds under the relevant experimental conditions, we also fit the equation for a second-order degradation of DCBQ to the data with DCBQ alone at both pHs to compare with the single-exponential (first order) case above. The fitted plots and residuals from the fits are detailed in Figure SN4.

$$\frac{1}{[DCBQ]} = \frac{1}{A} - 2kt$$

At pH 7, a second-order model yields a slightly better fit than at first-order, however the residuals in both cases are relatively low and normally-distributed. At pH 9.0, the fit to second order is significantly worse than that to a simple exponential. Therefore, the use of a pseudo-first-order scheme here is accurate at pH 9, and a reasonable approximation at pH 7.

We also assume a single "sink". This is an oversimplification as this term generically represents a complex pool of downstream species (such as the hydroxylated quinone derivatives and further oxidative degradation products that give rise to the additional absorbance peaks observed in the spectra), but the limited number of available datasets (DCBQ and $DCSQ_d$ peak currents) necessitates a minimal parameter model to reduce the risk of overfitting.

Beyond the four rate constants (k_1, k_{-1}, k_2, k_{-2}), constant offsets were also fitted to the DCBQ and $DCSQ_d$ traces to account for background contributions from other overlapping signals (Table SN2). The initial concentration of DCBQ was treated as a fitting parameter, while the initial concentrations of all other species were assumed to be zero.

At pH 9.0, the model fits the observed concentration curves well (Figure SN5), with low error on the rate constants. A full parameter fit (Table SN2) indicated that the values of k_{-1} and k_{-2} were within error of zero, and hence a simplified fit with the backward reactions eliminated was also carried out to obtain more reliable values of the forward rate constants k_1 and k_2.

At pH 7.0, the proposed differential equation model fits the observed concentration curves (Figure SN5). However, the associated errors remain high. This high uncertainty is largely attributed to the comparatively low values of k_1 and k_2, along with substantial experimental error in the determination of $DCSQ_d$ concentration. Notably, even though ferricyanide appears to increase the rate of the backward reaction, the high variability in the data complicates the extraction of a reliable steady-state concentration of DCBQ.

Table SN2. Obtained parameters from fitting of SWV data to the proposed model

Parameter	pH 7		pH 9		
	DCBQ	DCBQ + FeCN	DCBQ (simplified model)	DCBQ (full kinetic model)	DCBQ + FeCN
k_1	$(1.20 \pm 0.18) \times 10^{-2}$	$2.32 \times 10^{-3} \pm 11.60$	$(7.18 \pm 0.66) \times 10^{-2}$	$(7.26 \pm 1.09) \times 10^{-2}$	$(3.64 \pm 0.28) \times 10^{-2}$
k_{-1}		$3.15 \times 10^{-3} \pm 17.00$		$(6.49 \pm 65.04) \times 10^{-4}$	$(2.04 \pm 0.76) \times 10^{-2}$
k_2	$(3.47 \pm 6.35) \times 10^{-1}$	$1.68 \times 10^{-1} \pm 1.84$	$(4.40 \pm 2.64) \times 10^{-2}$	$(4.36 \pm 3.47) \times 10^{-2}$	$(1.01 \pm 0.24) \times 10^{-2}$
k_{-2}	$(2.76 \pm 5.03) \times 10^{-1}$	$3.64 \times 10^{-1} \pm 3.99$		$6.46 \times 10^{-20} \pm 0.01$	$(9.32 \pm 2.46) \times 10^{-3}$
y_0	235.82 ± 8.44	288.70 ± 7.20	188.79 ± 11.36	189.18 ± 12.43	307.18 ± 11.65
$Offset_1$	67.14 ± 8.26	119.85 ± 845050	43.84 ± 0.86	43.75 ± 1.34	$4.91 \times 10^{-14} \pm 29.60$
$Offset_2$	88.64 ± 12.84	92.02 ± 11.01	107.01 ± 14.74	106.76 ± 41.70	122.37 ± 10.16

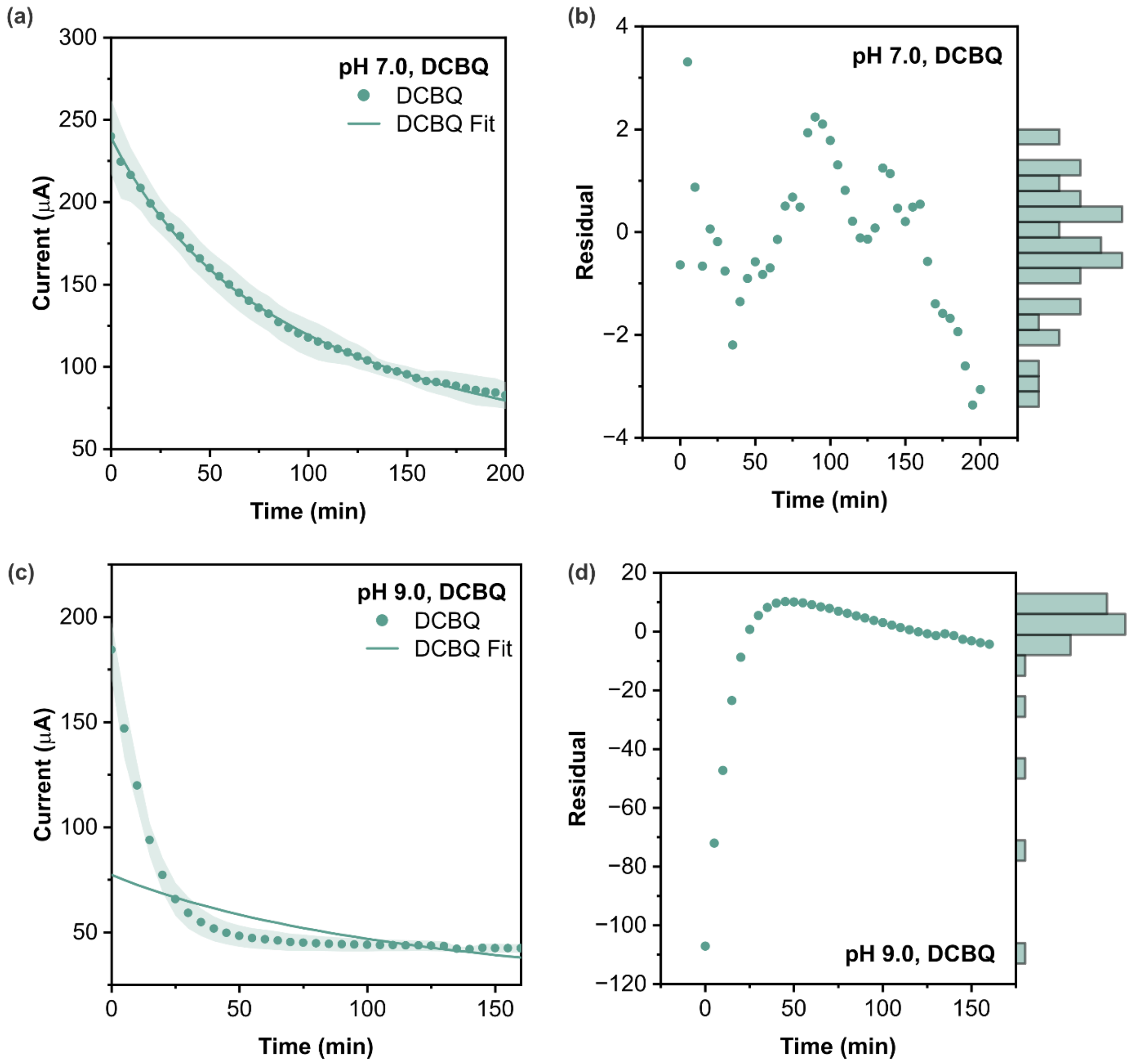

Figure SN4. Plots of DCBQ peak current over time at pH **(a)** 7.0 and **(c)** 9.0 along with their second-order exponential fittings and residual plots (**(b)** and **(d)**, respectively).

.

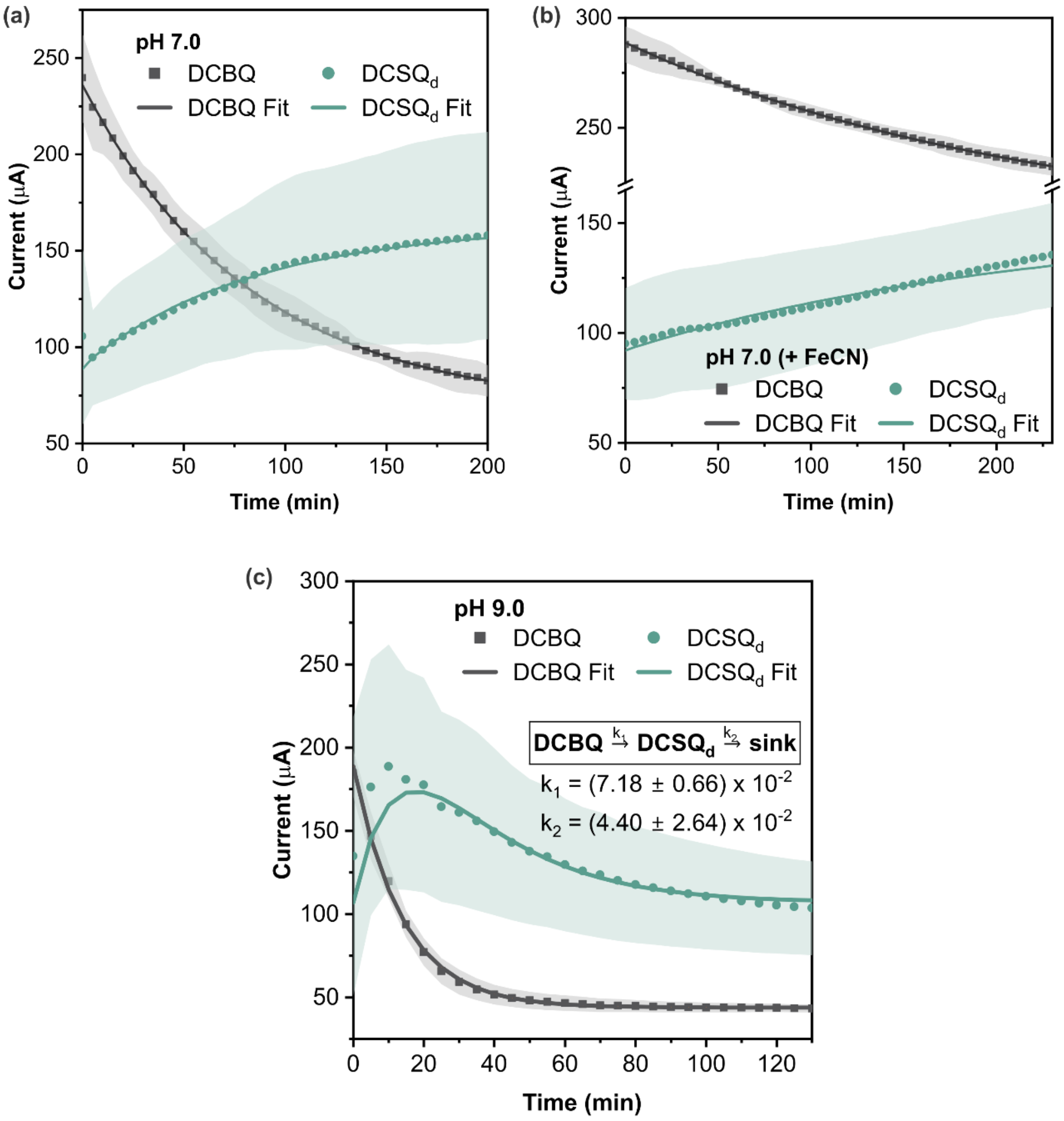

Figure SN5. Plots of experimental and predicted DCBQ and $DCSQ_d$ peak currents over time at pH 7.0 **(a)** without and **(b)** with ferricyanide, and **(c)** at pH 9.0 without ferricyanide along with their fittings to the proposed model.
